# Deoxycholic acid exacerbates intestinal inflammation by modulating interleukin-1*β* expression and tuft cell proportion in dextran sulfate sodium-induced murine colitis

**DOI:** 10.7717/peerj.14842

**Published:** 2023-02-15

**Authors:** Jingyi Ju, Cui Zhang, Jiaolan Yang, Qinglu Yang, Pengyun Yin, Xiaomin Sun

**Affiliations:** 1 Gastroenterology Department, The Shanghai Tenth People’s Hospital, Shanghai, People’s Republic of China; 2 Medical College, Tongji University, Shanghai, People’s Republic of China

**Keywords:** Inflammatory bowel disease, Deoxycholic acid, Secondary bile acids, IL-1β, Intestinal flora, Tuft cells

## Abstract

**Background:**

The etiology of inflammatory bowel disease (IBD) remains unclear. However, intestinal metabolism is known to be critical in the pathogenesis of IBD. Bile acid is one of the main intestinal metabolites, and its role in the pathogenesis of IBD is worthy of investigation. This study investigated the role of deoxycholic acid (DCA), a bile acid, in the pathogenesis of IBD.

**Methods:**

Peripheral serum metabolomics, fecal metabolomics, and microbiome analyses were performed on patients with IBD and healthy controls. Flow cytometry, real-time quantitative polymerase chain reaction, western blotting, enzyme-linked immunosorbent assay, immunohistochemical staining, and immunofluorescence analysis were used to evaluate cytokines in the inflamed colonic mucosa and immune cells and tuft cells in the intestine of mice with dextran sulfate sodium (DSS)-induced colitis.

**Results:**

In total, 156 patients with IBD and 58 healthy controls were enrolled. DCA levels in the serum and feces of patients with IBD were significantly decreased compared to the controls. This decrease was associated with a decrease in the abundance of intestinal flora, including Firmicutes, Clostridia, Ruminnococcaceae, and Lachnospiraceae. Additionally, interleukin (IL)-1*β* levels in the serum of patients with active Crohn’s disease were significantly increased compared with the healthy controls. Moreover, in DCA-treated DSS-induced mice, the expression of IL-1*β* and the proportion of CD3^+^ and CD4^+^ T cells increased while the number of intestinal tuft cells decreased, compared with the DSS group.

**Conclusion:**

In IBD patients, the decreased DCA levels in serum and fecal samples are associated with disturbances in gut microflora diversity and abundance. Possible mechanisms by which DCA affects immunity in DSS-induced murine colitis include increasing IL-1*β* secretion, reducing the number of tuft cells in the mucosa, and activating CD4^+^ and CD3^+^ T cells to exaggerate immune responses, consequently worsening intestinal inflammation.

## Introduction

Inflammatory bowel disease (IBD), which includes Crohn’s disease (CD) and ulcerative colitis (UC), is a chronic inflammatory disease of the gastrointestinal tract. CD may involve all layers of the intestinal tract and its main clinical manifestations include recurrent abdominal pain, diarrhea, intestinal stenosis, perianal abscesses, as well as intestinal and anal fistulae (Torres et al., 2017). Intestinal inflammation in patients with UC is limited to the colonic mucosa, which mainly manifests as abdominal pain; stools containing mucus, pus, and blood; and susceptibility to complications due to toxic megacolon (Ungaro et al., 2017).

IBD is a highly disabling diseases and its patients are prone to relapse. Not only does IBD affect the health of patients, but it also puts a huge economic pressure worldwide (Kaplan, 2015). The occurrence and progression of IBD are linked to many factors, including genetic factors, microbial diversity, immunity, the intestinal mucosal barrier, and the environment (Ramos & Papadakis, 2019). Therefore, seeking a new, effective, and safe treatment is important for IBD.

Trillions of microorganisms populating the human intestine contribute in various biological and immune processes in the host (Ni et al., 2017). In this regard, intestinal inflammation was found to be associated with a decrease in the abundance and diversity of intestinal flora. For instance, in these conditions, an increase in Proteobacteria and decrease in Firmicutes abundance are observed (Liguori et al., 2016; Manichanh et al., 2006). Additionally, dysbiosis in the intestinal flora was reported to be more significant in active IBD (Duboc et al., 2013).

Metabolomics is a research tool for quantitatively analyzing metabolites in organisms and examining the relationship between metabolites and physiological or pathological changes. In recent years, bile acid (BA) metabolites, tryptophan metabolites, and short-chain fatty acids were found to play important roles in the regulation of inflammation in IBD (Lavelle & Sokol, 2020). Specifically, the relationship between BA metabolites and IBD has attracted increasing attention.

BAs mainly function by activating receptors to regulate glucose, lipid, and energy metabolism (Jiao et al., 2018; Xie et al., 2021). Primary BAs are derived from cholesterol in the liver and are combined with either glycine or taurine to synthesize conjugated BAs, which, in turn, are stored in the gallbladder and transported to the intestine when meals are ingested. Most conjugated BAs are reabsorbed in the terminal ileum, while others are further converted to secondary BAs by colonic microbiota and eventually excreted in feces (De Aguiar Vallim, Tarling & Edwards, 2013). Since the intestinal microbiota contributes to BA synthesis and transformation (Guzior & Quinn, 2021), dysbiosis of the microbiome leads to a decrease in uncoupling and 7 *α*-dehydroxylation, resulting in a decrease in secondary BA levels. BA metabolism disorders affect immunity and the intestinal barrier function (Thomas et al., 2022). For instance, under the influence of diet and the gut microbiota, intestinal BA metabolites increase the number of Foxp3^+^ regulatory T cells (Treg) in the lamina propria. This increase leads to Treg cell differentiation, which inhibits Th17 cell differentiation, ultimately alleviating intestinal inflammation (Campbell et al., 2020; Hang et al., 2019; Song et al., 2020). Furthermore, BAs promote the regeneration and differentiation of epithelial cells by activating receptors on the intestinal stem cells to repair the intestinal epithelium (Fu et al., 2019; Sorrentino et al., 2020). Thus, BAs play a key role in the regulation of intestinal immunity and homeostasis. Thus, regulatin g BAs has great potential for improving enteritis.

Deoxycholic acid (DCA) can affect the total number and diversity of intestinal flora, increase the abundance of Firmicutes, and decrease the abundance of BA-sensitive Bacteroidetes (Yokota et al., 2012). In contrast, DCA has been shown to decrease intestinal stem cell function *via* endoplasmic reticulum stress (Huang et al., 2020). Additionally, DCA can act directly on the vascular endothelium, increasing the expression levels of the adhesion molecules ICAM-1 and VCAM-1 *via* S1PR2. This phenomenon results in increased lymphocyte movement in the small intestine, consequently modifying the intestinal immunological milieu (Shibuya et al., 2021). Moreover, DCA was found to delay wound healing in mouse colonic epithelial wounds by activating AKT (Azuma et al., 2021).

In this study, we hypothesized that DCA levels in patients with IBD are lower than those in healthy individuals. Additionally, we aimed to identify the possible mechanism by which DCA affects intestinal inflammation and to uncover new targets for the treatment of patients with IBD. Taken together, our results shed lights on the effects that alteration of the gut bile acid level, such as DCA, may have on the gut microbiota and intestinal inflammation. These findings may be of pivotal importance in clinical treatment of IBD.

## Materials & Methods

### Reagents

All the reagents used are listed in Table S1.

### Subjects

Patients diagnosed with CD and UC were recruited from the department of gastroenterology, while healthy patients (control) were recruited from the center of physical examination at Shanghai Tenth People’s Hospital of Tongji University (Shanghai, China). Crohn’s Disease Activity Index (CDAI) score was used to evaluate CD activity, and a CDAI score >150 was considered to indicate active CD (Best et al., 1976).

UC activity was measured using the Mayo score, which ranges from 0 to 12 (0 = no disease, and 12 = most severe disease) (Rutgeerts et al., 2005). Patients with cholecystitis, pancreatitis, liver disease, malignant tumors, recent infectious diseases, organ dysfunction, or a history of intestinal surgery were excluded. All the participants provided written informed consent. All procedures and experiments were approved by the Human Ethics Committee of the Shanghai Tenth People’s Hospital (No.: SHSY-IEC-BG-05.08/05.0 22KN187).

### Human IL-1*β*, IL-17A, IFN-*γ*, TNF-*α*, IL-6, IL-8, and IL-4 ELISA

Serum was collected from the patient’s blood, after centrifugation at 1000 g for 20 min. IL-Than IL-1*β*, IL-17A, IFN-*γ*, TNF-*α*, IL-6, IL-8, and IL-4 were detected using ELISA according to the manufacturer’s instructions.

### Targeted metabolomics analysis of serum and fecal samples

Add methanol (0.75 mL) to a 100 mg or 100 ul sample, place it in a glass tube with a polytetrafluoroethylene lined cover, and vortex the tube. Add 2.5 ml MTBE and incubate the mixture on an oscillator at room temperature for 1 h. Phase separation was induced by adding 0.625 mL of MS grade water. After incubation at room temperature for 10 min, the sample was centrifuged at 1000 g for 10 min. Collect the upper (organic) phase and re extract the lower layer with one mL of solvent mixture (MTBE/methanol/water (10:3:2.5, v/v/v)) to collect the upper phase. Dry the combined organic phase and dissolve it in 100 µL in isopropyl alcohol. Then analyze by LC-MS/MS. The Biozeron Biotech Vanquish UHPLC system (Thermo Fisher, Dreieich, Germany) and the Orbitrap Q ExecuteTM HF mass spectrometer (Thermo Fisher, Dreieich, Germany) were used for UHPLC-MS/MS analysis.

### Microbial diversity analysis

Microbial DNA was extracted from human fecal samples using the Omega (EZNA) Stool DNA Kit (USA). Bacterial 16S ribosomal RNA gene in the V1–V9 region was amplified using PCR (95 °C for 2 min, followed by 27 cycles at 95 °C for 30 s, 55 °C for 30 s, and 72 °C for 60 s, and a final extension at 72 °C for 5 min). The following primers used: 27 F 5′-AGRGTTYGATYMTGGCTCAG-3′ and 1492 R 5′-RGYTACCTTGTTACGACTT-3′.

PCR was performed in triplicates, in 20 µL mixtures containing 4 µL 5 FastPfu Buffer, 2 µL 2.5 mM dNTPs, 0.8 µL primers (5 µM), 0.4 µL FastPfu polymerase, and 10 ng template DNA. Amplicons were extracted from 2% agarose gels and purified using an AxyPrep DNA Gel Extraction Kit.

### Mice

Eight-week-old female C57BL/6J mice, weighing 16–18 g were obtained from Shanghai SLAC Laboratory Animal Co., Ltd. (Shanghai, China). Briefly, mice were maintained on a 12-h light/dark cycle. The mice were housed in ventilated cages containing three to seven mice per cage and were fed sterilized food and water.

### Colitis induction and DCA enema

The mice were divided into four groups: drinking water and water enema (water + water); drinking water and DCA enema (water + DCA); drinking dextran sulfate sodium (DSS) and water enema (DSS + water); and drinking DSS and DCA enema (DSS + DCA). DSS was dissolved in drinking water to 2.5% (w/v) and was consumed by the mice beginning on day 0, for 7 days. All mice were provided drinking water on days 8–10. Mice were sacrificed by cervical dislocation on day 11. Mice were anesthetized before the enema using 4% chloral hydrate prepared in saline.

To prepare DCA suspension, 5 mg DCA was mixed in 150 µL water and sonicated. DCA suspension or water was instilled in the rectum for at least 3 min on days 4, 6, and 8. Mice were sacrificed on day 11. The colonic mucosa was collected, and the length of the colon was measured. The raw data of the study are provided in the Supplementary material: raw data. The animal study was approved by the Ethics Committee of the Shanghai Tenth People’s Hospital (No.: SHDSYY-2021-3292).

### Hematoxylin and eosin (H&E) staining and assessment of colitis

The distal segments of the colon were harvested, and a piece of colon tissue was fixed overnight with 4% paraformaldehyde solution and embedded in paraffin before cuting into 4 µm slices. After baking slices at 65 °C for 1.5 h, the slices are dewaxed in xylene and rehydrated in gradient alcohol. Then samples were then processed for paraffin sectioning and stained with H&E (C015; Beyotime, Shanghai, China), the sections were observed under a light microscope, and histopathological scores were assessed for each mouse. A detailed description of these scores is provided in Table S2.

### Immunofluorescence staining

Slides were deparaffinized by incubating in xylene for 15 min, for a total of two washes. Slides were then dehydrated with absolute ethanol for 5 min, twice. Next, they were dehydrated sequentially in 85% and 75% ethanol for 5 min each. The slides were washed with distilled water, and antigen retrieval was performed in a buffer containing EDTA (pH 8.0). The temperature was maintained at sub-boiling for 8 min, standing for 8 min, and another sub-boiling for 7 min. The samples were washed thrice with PBS (pH 7.4) for 5 min each in a Rocker device (TSY-B; Servicebio), and the excess fluid was removed. The sample was marked using a liquid-blocking pen. To prevent nonspecific binding, samples were incubated in 3% BSA for 30 min. Samples were then incubated with the following primary antibodies at 4 °C overnight: cyclooxygenase 2 (COX-2), phosphorylated epidermal growth factor receptor (p-EGFR), and doublecortin and CaM kinase-like-1 (DCAMKL-1) from Abcam. The slides were washed three times with PBS for 5 min each and then incubated with a secondary antibody in the dark at room temperature for 50 min. The slides were washed as described above and incubated with DAPI for nuclear staining at room temperature for 10 min in the dark.

### Immunohistochemical staining

Immunohistochemical staining was performed to evaluate the expression of IL-1*β* using an IL-1*β* antibody according to the manufacturer’s protocol. The slides were scanned and observed under a Leica Microsystems optical microscope (Danaher, Washington D.C., USA).

### Real-time quantitative polymerase chain reaction (RT-qPCR)

Total RNA was extracted from the samples using the EZ-press RNA Purification Kit (EZBioscience, Roseville, MN, USA), and the concentration was estimated using a NanoVue spectrophotometer (GE Healthcare, Chicago, IL, USA). The PrimeScriptTM RT Reagent Kit (Applied Biological Materials, Richmond, Canada) was used to synthesize cDNA. The following conditions were used: 25 °C for 10 min, 42 °C for 15 min, and 85 °C for 5 min. RT-qPCR was performed with SYBR Green using the RT-qPCR platform LightCycler 96 (Roche, Basel, Switzerland). Thermocycling conditions for RT-qPCR were according to the protocol: 95 °C for 5 s and 60 °C for 34 s, repeated for 40 cycles. Primers for the genes tumor necrosis factor alpha (*Tnf- α*), interferon gamma (*Ifn- γ*), interleukin-17 A (*Il-17a*), *Il-1β*, *Il-4*, *Il-6,* and *Il-8* were obtained from BGI Genomics (Shenzhen, China). The primer sequences are listed in Table S3. Data were analyzed by calculating the 2 − Δ ΔCt relative fold-change.

### Flow cytometry

Intestinal lamina propria mononuclear cells (LPMCs) were isolated from mouse colon tissues (Wu et al., 2022). For fluorescence-activated cell sorting (FACS) analysis, isolated cells were suspended in FACS buffer, and single-cell suspensions were stained with the following antibodies: APC anti-mouse CD3, FITC anti-mouse CD4, PE anti-mouse CD8, PE/Cy7 anti-mouse B 220, APC anti-mouse CD11b, FITC anti-mouse F4/80, PerCP-cy5.5 anti-mouse Ly6C, PE/Cy7 anti-mouse Ly6G, APC anti-mouse CD11b, PE anti-mouse CD11c, and FITC anti-mouse MHC II. Cells were incubated with primary antibodies at 4 °C for 30 min. Cell subtypes were analyzed using LSR II (BD Biosciences, Franklin Lakes, NJ, USA), and data were processed using Flow Jo 10.0 (Tree Star, Ashland, OR, USA) software.

### Murine IL-1*β* ELISA

Colon samples (0.5 cm) from mice were cultured in 24-well tissue culture plates containing one mL RPMI 1640 medium with L-glutamine and phenol red overnight at 37 °C. Culture supernatants were collected, and ELISA was performed with the supernatants according to the manufacturer’s instructions to quantify IL-1*β*.

### Western blotting

Total protein was extracted from ground colon tissues using RIPA lysis buffer containing protease inhibitors. Protein concentrations were measured using a BCA kit. The proteins were separated using SDS-PAGE and transferred to a PVDF membrane (0.22 µm). The membrane was blocked with 5% skimmed milk for 1 h at room temperature. Next, the membrane was incubated with primary antibodies against IL-1*β* (dilution 1: 500) and *β*-actin (dilution 1: 20000) overnight at 4 °C. The membrane was washed with diluted TBST buffer three times for 5 min each. The corresponding secondary antibody (SA00001-1, SA00001-2) was then incubated with the primary antibody for 1 h at room temperature. The blots were then washed again with TBST. Chemiluminescence was used to visualize the protein blots using an Enhanced Pico Light chemiluminescence kit and the Chemiluminescence Imager (Amersham Imager 600; Amersham, Woburn, MA, USA).

### Statistical analysis

Results are expressed as the mean ± SEM from three independent experiments. Mean differences between two groups were analyzed using unpaired, two-tailed, Student’s *t*-test. Variables with skewed distributions were expressed as medians and interquartile ranges, and comparisons between groups were performed using nonparametric tests. Pairwise comparisons between multiple groups were performed using the Bonferroni correction. Correlations between BA level and intestinal flora abundance were assessed using Spearman’s correlation coefficient. Results were considered significant statistically when *P* < 0.05 (*), *P* < 0.01 (**), and *P* < 0.001 (***).

## Results

### General information on IBD patients and healthy controls

From November 2020 to June 2021, patients with IBD and controls who met the inclusion criteria included 60 patients with CD in remission (R-CD), 66 patients with active CD (A-CD), and UC patients in remission (R-UC, 4 cases), active UC (R-UC, 26 cases), and healthy controls (HC, 58 cases) (Table 1).

**Table 1:** General information on IBD patients and healthy controls.

Variable		CD (*n* = 126)	UC (*n* = 30)	IBD (*n* = 156)	HC (*n* = 58)	*P* value
Age		37.39 ± 15.12	42.63 ± 17.24	38.40 ± 15.63	55.59 ± 14.06	0.078
Gender	Male	90 (71.43%)	21 (70.00%)	111 (71.15%)	30 (51.72%)	
	Female	36 (28.57%)	9 (30.00%)	45 28.85%)	28 (48.28%)	
Course of Disease (month)		43 (1, 576)	24 (1, 144)	36 (1, 576)	–	
CDAI		135.62 ± 101.79	–			
Mayo Score		–	7.50 ± 3.93			

### Serum DCA levels were decreased in IBD patients

**Figure 1:**
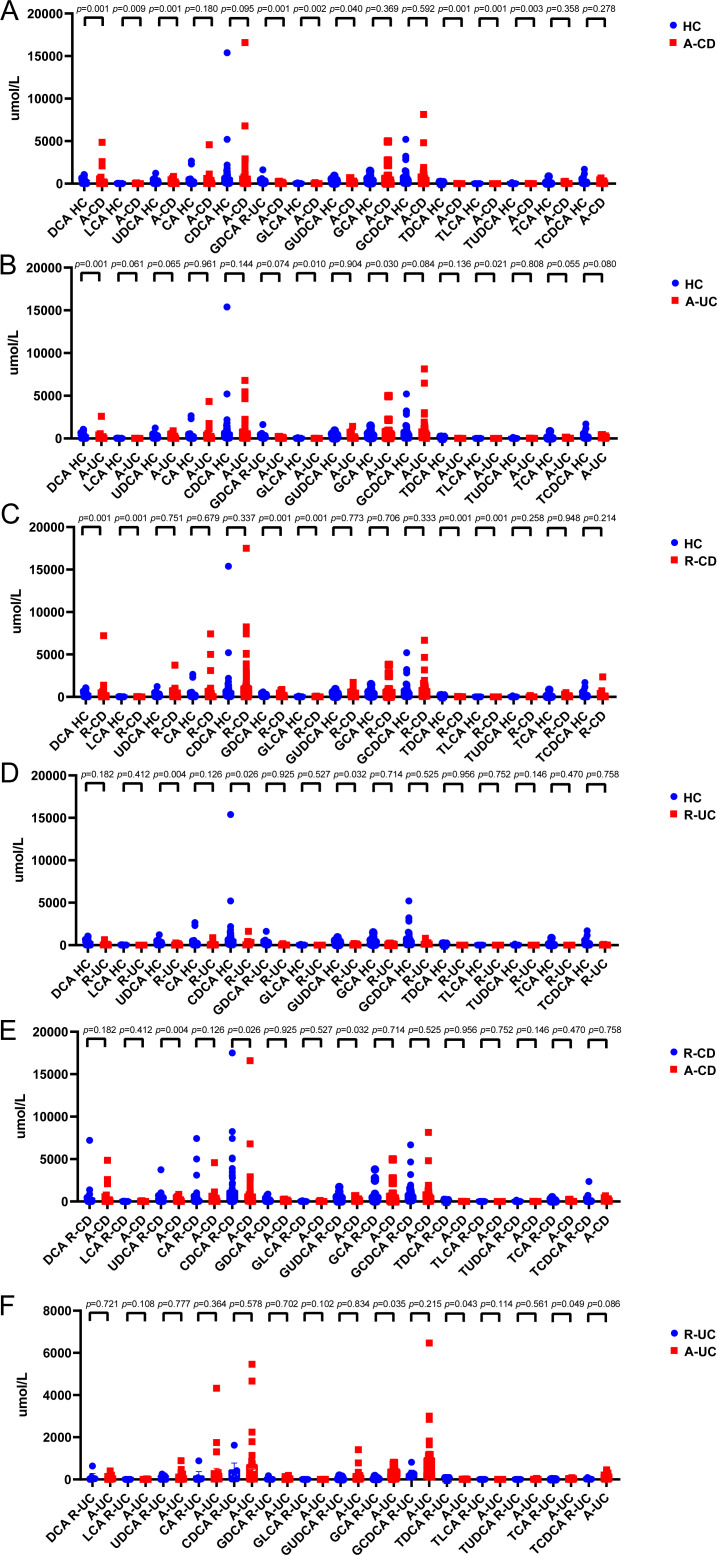
Secondary bile acids decrease in the serum of patients with IBD. (A–D) Levels of DCA, LCA, UDCA and their combination with glycine and taurine in IBD patients were lower, and the levels in the CD patients in active phase was significantly lower. (E). The level of UDCA was significantly lower in the CD active period than in the remission period. (F). The bile acids were not significantly different between CD patients in active phase and in remission. (HC *n* = 58, CD-Remission *n* = 60, CD-Active *n* = 66, UC-Remission *n* = 4, UC-Active *n* = 26).

The serum levels of DCA, lithocholic acid (LCA), Glycodeoxycholic acid (GDCA), Glycolithocholic acid (GLCA), taurodeoxycholic acid sodium salt (TDCA), and Taurolithocholic acid sodium salt (TLCA) in patients with CD in remission were significantly lower than those in healthy controls. The serum levels of DCA, LCA, Ursodeoxycholic acid (UDCA), GDCA, GLCA, Glycoursodeoxycholic acid (GUDCA), TDCA, TLCA, and Tauroursodeoxycholic acid Dihydrate (TUDCA) in patients with active CD were significantly lower than those of healthy controls. Additionally, the level of serum UDCA in patients with remission CD was higher than that in active CD (Fig. 1).

### Fecal DCA levels were decreased in IBD patients

**Figure 2:**
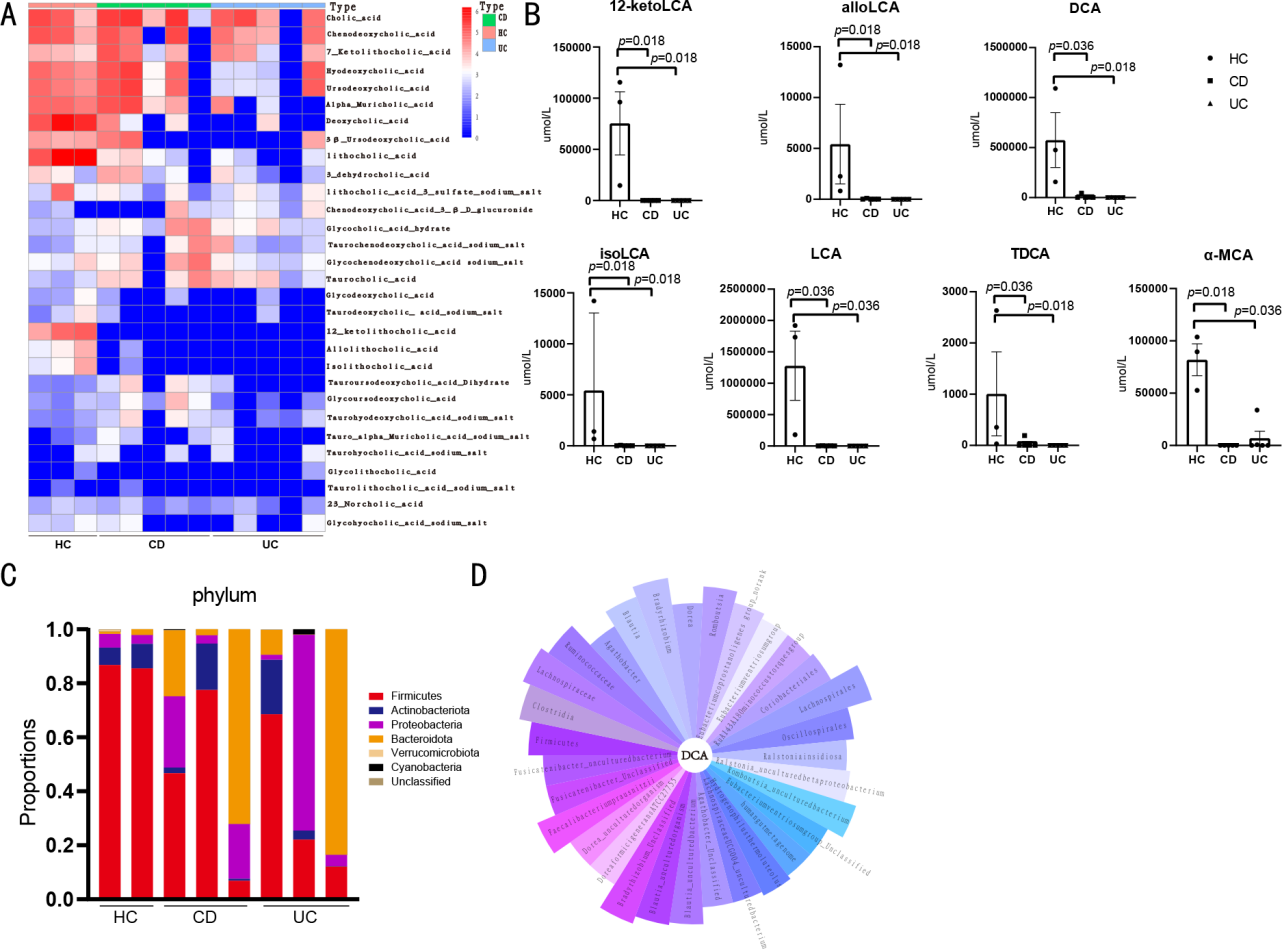
Fecal bile acids are associated with intestinal flora. (A) Heatmap of human fecal bile acids. (HC *n* = 3, CD *n* = 5, UC *n* = 5). (B) Compared with healthy controls, levels of 12-ketoLCA, alloLCA, DCA, isoLCA and LCA in feces of patients with CD and UC were significantly lower. Level of TDCA was lower in CD patients. Level of *α*-MCA significantly lower in UC patients compared with healthy controls. (C) The abundance at phylum level: the abundance of Firmicutes decreased in patients with CD and UC. (D) The petal plot shows the intestinal flora associated with DCA, with the band length representing the magnitude of the absolute r of the correlation coefficient.

The levels of 33 fecal BAs and metabolites were measured using targeted metabolomic analysis (Fig. 2A). The 12-ketolithocholic acid (12-ketolca), allolithocholic acid (alloLCA), DCA, isolithocholic acid (isoLCA), LCA, and TDCA levels were significantly decreased in patients with CD compared with healthy controls. Moreover, 12-ketolca, allocholic acid, DCA, isoLCA, LCA, and Alpha-Muricholic acid (*α*-MCA) levels were significantly decreased in patients with UC when compared with healthy controls (Fig. 2B).

### Intestinal flora diversity and abundance were aberrant in IBD

Microbiome analyses of the feces of patients and healthy controls were performed (Fig. 2C). The abundance of Firmicutes and Clostridia in samples from patients with CD and UC was lower than that in healthy controls. Moreover, in patients with CD, the abundance of Lachnoclostridium and Microbacterium was significantly higher than that in the controls, but *Bacteroides ovatus* abundance was lower than that in the controls. As for patients with UC, the abundance of Intestinibacter was lower than that in controls. Moreover, the abundance of Microbacteriaceae was lower and the abundance of Bacillus was higher in patients with UC than that in patients with CD.

### BA levels in feces correlated with intestinal flora

A correlation analysis between fecal BA levels and intestinal flora is shown in Fig. 2D. The fecal DCA may be correlated with Firmicutes, Clostridia, Lachnospiraceae, and Ruminococcaceae abundance. Clostridia were strongly correlated with DCA, and the abundance of Clostridia was significantly lower in patients with CD and UC than that in healthy controls.

### Colonic inflammation was exacerbated by DCA in DSS-induced acute colitis

**Figure 3:**
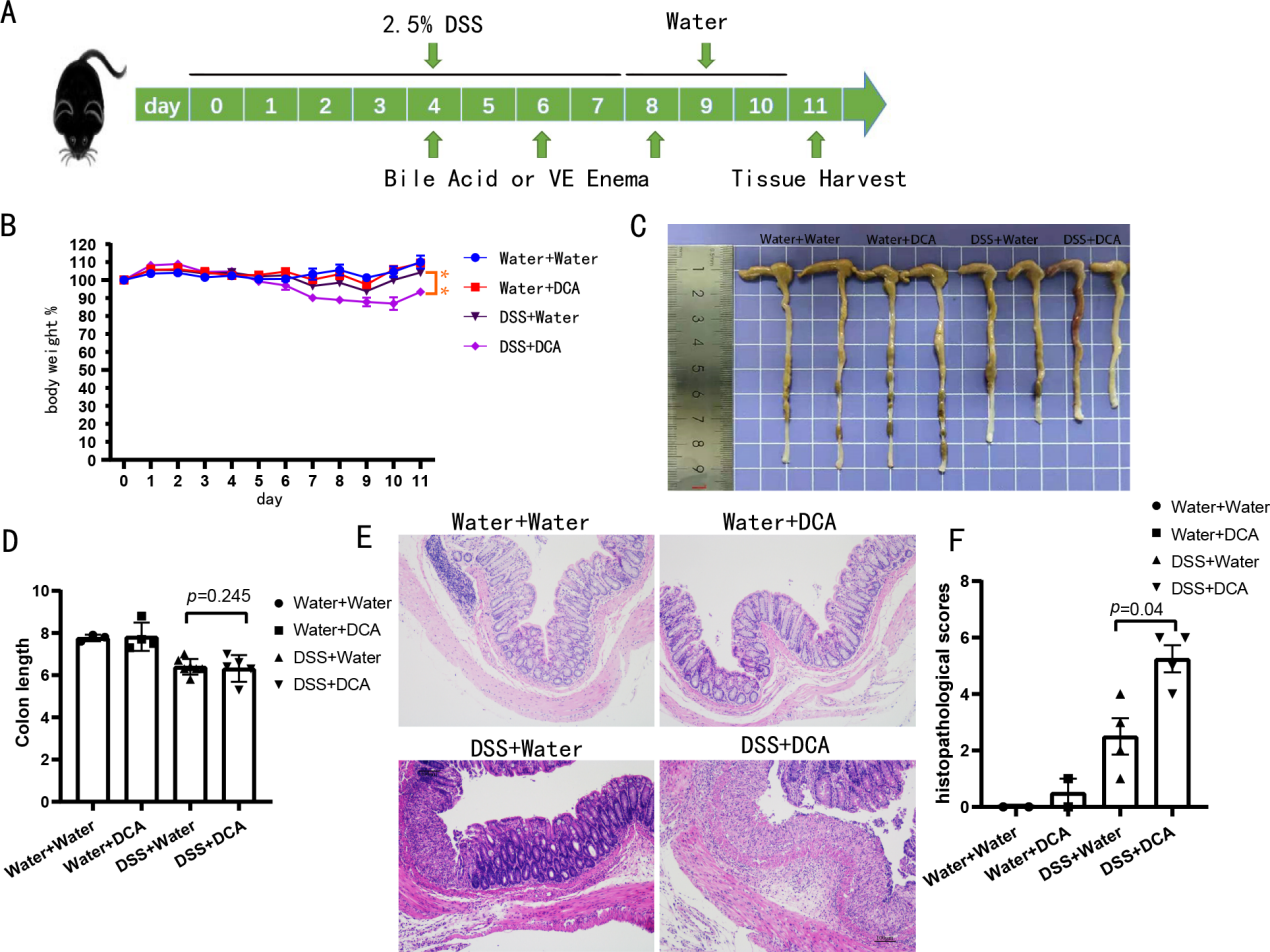
DCA aggravated the DSS-induced enteritis model. (A) C57BL/6 mice were given water containing 2.5% DSS (w/v) for 7 days and treated with suspension of bile acid or vehicle control (VE) per rectum on days 4, 6, and 8 with 150 uL of either, 5 mg of DCA. (B–C) DCA treatment promoted enteritis, with lower body weight (B) and more colon inflammation (C). (D) Compared with DSS group, DCA combination group mice had shorter colon length. (E) In the DCA combination group, the colonic crypts are damaged, inflammatory cells infiltration and mucosal erosions are more serious (Scale bar, 100 um) (F) DCA increased the histopathological scores.

The mice were divided into four groups: water + water (*n* = 3), water + DCA (*n* = 4), DSS + water (*n* = 7), and DSS + DCA (*n* = 7). DCA was administered to the mice *via* enema (Fig. 3A). DCA did not reduce intestinal inflammation. In the combined treatment group (DSS + DCA), two mice died on days 9 and 11. The combined treatment group lost more weight (Fig. 3B), showed more noticeable colonic mucosa edema, and had a shorter colon length than the DSS + water group (Figs. 3C and 3D). H&E staining (Fig. 3E) revealed severe damage in the colonic crypts of the DCA group. Moreover, mucosal erosions were more serious, and inflammatory cell infiltration was more evident in the combined treatment group. Additionally, DCA increased the histopathological scores (Fig. 3F).

### IL-1*β* expression and the proportion of CD3^+^ and CD4^+^ T cells were increased by DCA in DSS-induced acute colitis

**Figure 4:**
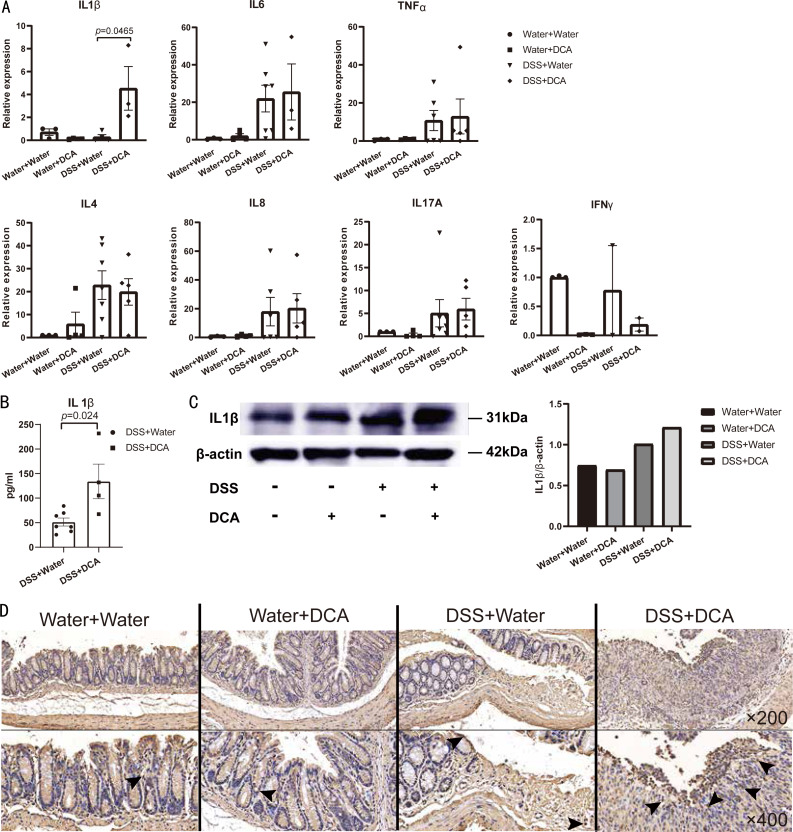
The expression of IL-1 *β* in colonic epithelium was higher in DCA combination group compared with the DSS. (A) qRT-PCR: The expression of IL-1 *β* in colonic epithelium was increased in DCA group compared with DSS group. (B–C) The difference in the expression of IL-1 *β* was increased in DCA combination group compared with DSS group in ELISA (B), and Western Blot (C). (D and E) Representative immunohistochemical staining profiles of IL-1 *β* in colonic tissues at ×200 magnification. Scale bar = 20 µm and at × 400 magnification. Scale bar =50 µm.

**Figure 5:**
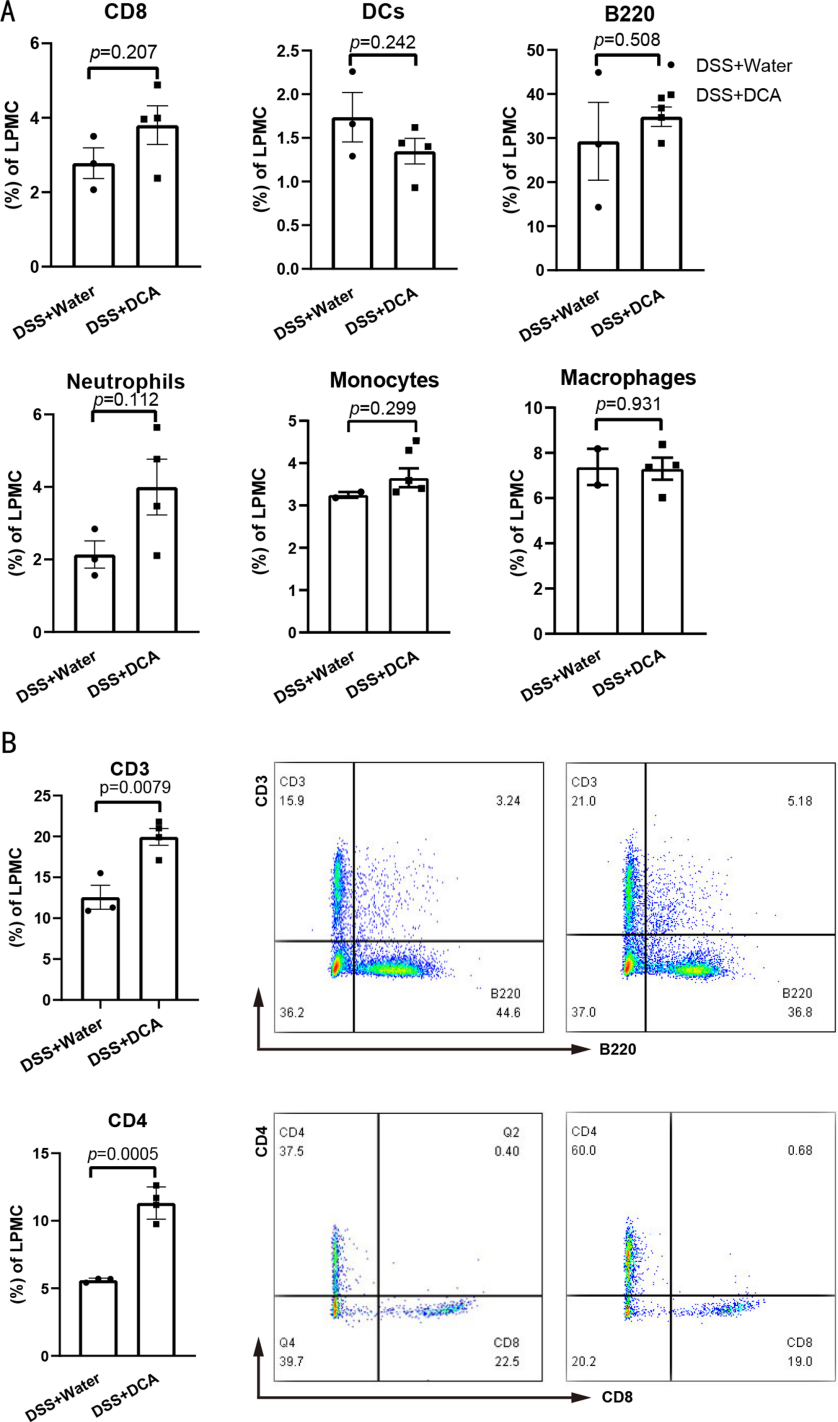
The proportion of CD3^+^ and CD4^+^ T cells were higher in DCA combination group. (A) There was no difference in the number of CD8^+^ T cells, B cells, neutrophils, monocytes, macrophages and dendritic cells (DCs) among the intestinal lamina propria mononuclear cells (LPMC) between DSS group and the combination group. (B). Compared with DSS group, the proportion CD3^+^T lymphocytes and CD4^+^ helper T lymphocytes increased in the combination group in LPMCs.

RNA from the distal colon tissues of mice showed that DCA increased the expression of *Il-1β* in colonic mucosa (Fig. 4A). Significant differences in the expression of IL-1*β* between DSS- and DCA-treated groups were verified using ELISA (Fig. 4B) and western blotting (Fig. 4C). Moreover, flow cytometric analysis of mouse LPMCs showed no difference in the proportion of macrophages, dendritic cells, monocytes, and neutrophils (Fig. 5A), whereas the proportion of CD3^+^ and CD4^+^ T cells in the combined treatment group was higher than that in the DSS group (Fig. 5B).

### Proteobacteria abundance increased in the DCA-treated DSS-induced colitis

**Figure 6:**
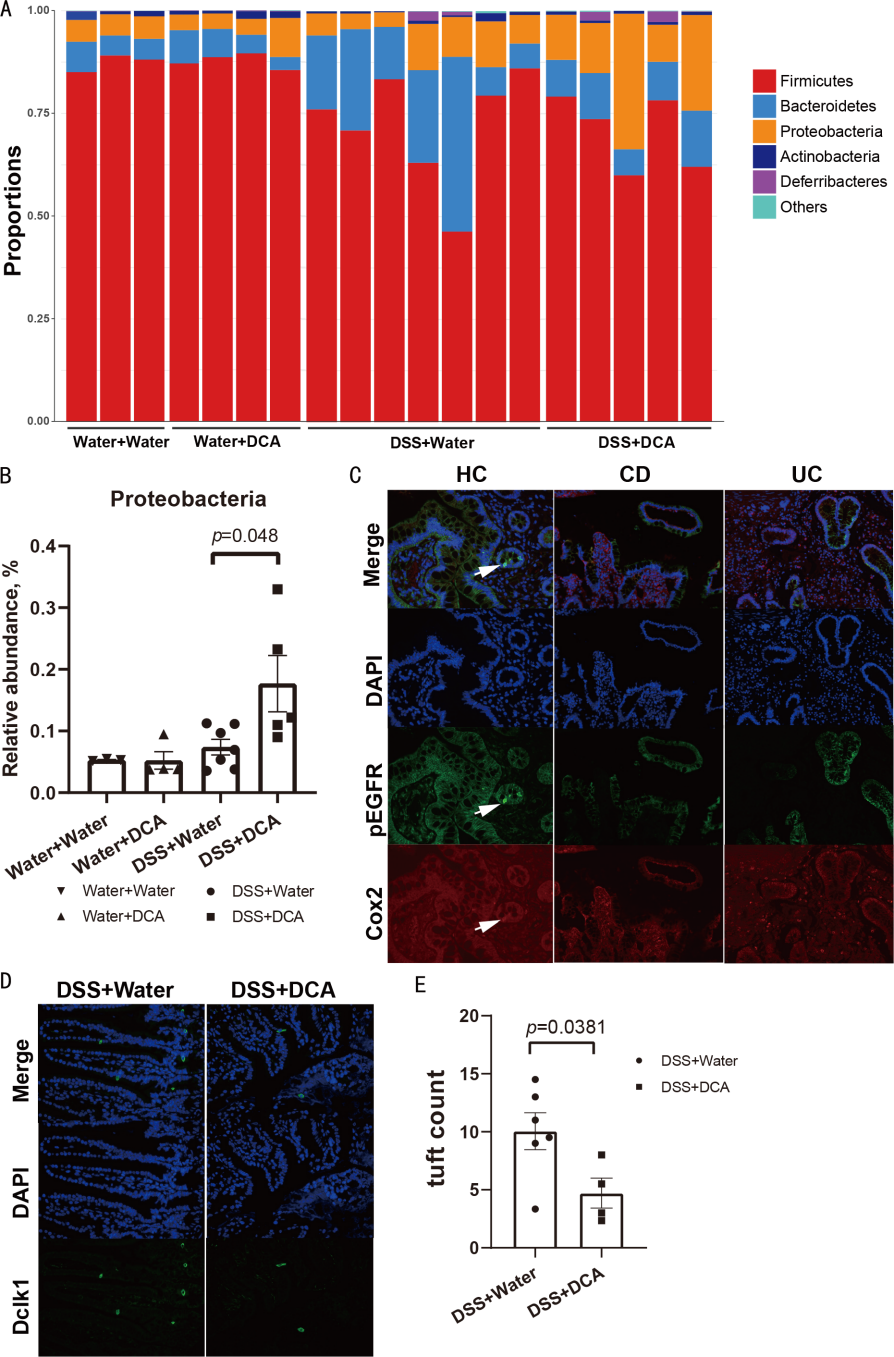
The relative abundance of Proteobacteria was increased in the combination group and tuft cells decreased in the small intestine of the combination group. (A) The abundance of microflora at the phylum level among the groups. (B) The relative abundance of Proteobacteria between the DSS group and the combination group. (C) The tuft cells of the colon in healthy controls were few (original magnification ×200). (D) Compared with the DSS group, tuft cells in the small intestine of mice in the DCA group decreased (original magnification ×400). (E) Tuft cells in the colon of mice decreasd in the DCA group.

The microflora in the four groups are shown at the phylum level (Fig. 6A). The relative abundance of Proteobacteria was increased in the combined treatment group (*P* = 0.048) (Fig. 6B). However, the abundance of Clostridia, Lachnospiraceae, and Ruminococcaceae, which was linked to DCA levels in human feces, showed no significant difference between the DSS and combined treatment groups in mice.

### Number of tuft cells decreased with DCA treatment in DSS-induced acute colitis

We analyzed the number of tuft cells by immunofluorescence to monitor the differentiation of intestinal stem cells after BA intervention and intestinal mucosal barrier damage. COX-2 and p-EGFR were expressed in tuft cells in the intestinal tissues of humans obtained by colonoscopy biopsy, whereas DCAMKL-1 was expressed in tuft cells of mice. Immunofluorescence results suggested the presence of a few tuft cells in the colon of patients with IBD when compared with controls (Fig. 6C). The number of tuft cells marked by the antibody DCAMKL-1 was also decreased in the small intestine of the combined treatment group (Figs. 6D and 6E).

### Cytokine levels were increased in IBD patients

IL-1*β*, IL-17 A, IFN-*γ*, TNF-*α*, IL-6, IL-8, and IL-4 were detected using ELISA (Fig. S1). IL-1*β* and IFN-*γ* levels in sera from patients with active CD were significantly higher compared with the control. Moreover, IL-6 and IL-8 levels in the sera of patients with active CD and UC were significantly increased compared with the control group.

## Discussion

Studies have found that secondary BA levels in IBD patients are significantly lower than those in healthy individuals, which may be associated with changes in the gut microbiota (Lucas et al., 2021). In this study, serum BA levels were assessed in patients with IBD and healthy controls who met the inclusion criteria. The results showed that the levels of DCA, LCA, UDCA, and their conjugated BAs with glycine and taurine in the serum of IBD patients were significantly reduced, especially in the active phase of CD, compared with controls (Fig. 1). Additionally, BA levels differed between IBD patients with different disease activities, with lower UDCA levels in patients with active CD compared with those in patients in remission. This finding suggests that serum BA levels, especially UDCA levels, may serve as indicators for assessing disease activity in IBD.

Since abnormal BA metabolism can affect the levels of intestinal BAs, we examined fecal BA levels in patients (Fig. 2A). Our results revealed that fecal 12-ketoLCA, alloLCA, DCA, isoLCA, LCA, and TDCA levels were significantly reduced in CD patients, while fecal 12-ketoLCA, allocholic acid, DCA, isoLCA, LCA, and TDCA levels were significantly reduced in UC patients, compared with healthy controls. Moreover, *α*-MCA level in UC patients was significantly reduced compared with healthy controls (Fig. 2B), similar to the results of a previous study (Lucas et al., 2021). Most current studies have shown that reduced bile acid levels in IBD patients are associated with bile acid diarrhea, especially in patients with ileal Crohn’s disease and ileal surgery, and BA sequestrants are most commonly used in diagnosis and therapy (Camilleri, 2015).

Microbiome analysis results showed that the abundance of Firmicutes in patients with IBD was lower than that in controls (Fig. 2C), whereas the abundance of Bacteroides was higher, consistent with other studies (Manichanh et al., 2006). A possible correlation exists between fecal DCA levels and the abundance of microbes such as Firmicutes, Clostridia, Lachnospiraceae, and Ruminococcaceae (Fig. 2D). However, the composition of the dominant flora did not change significantly. In addition, the abundance of Lachnoclostridium and Microbacterium in patients with CD was higher than that in controls, while the abundance of *Bacteroid* es *ovatus* was lower than that in controls. Lachnoclostridium contains the bacterial marker m3, which can be used for non-invasive diagnosis of colorectal adenomas (Liang et al., 2020). As UC is known to be a risk factor for colorectal cancer, monitoring Lachnoclostridium levels may be important in assessing the disease in patients with UC. *Bacteroides ovatus* can increase the concentration of the tryptophan metabolite indole-3-acetic acid and stimulate dendritic cells to secrete the repair cytokine IL-22 to alleviate colon inflammation (Ihekweazu et al., 2021). Therefore, the significantly decreased abundance of *Bacteroides ovatus* in CD may have affected the intestinal repair function.

We found that fecal DCA may be related to various flora, including Firmicutes, Clostridia, Ruminococcaceae, and Lachnospiraceae, all of which can perform 7 *α*-dehydroxylation. Dysregulation of the bacterial community may cause a decrease in uncoupling and 7 *α*-dehydroxylation, leading to a decrease in secondary BA levels (Lucas et al., 2021). Lachnospiraceae and Ruminococcaceae belong to the phylum Firmicutes and are beneficial bacteria in the intestinal tract. Lachnospiraceae is the main bacterial family producing short-chain fatty acids (Sun et al., 2017), which play a key role in alleviating intestinal inflammation. Therefore, the decrease in Lachnospiraceae abundance is likely to affect intestinal inflammation. Moreover, we found that the abundance of Clostridia was significantly lower in patients with CD and UC than in controls, and a strong correlation was found between Clostridia and DCA in the correlation analysis. It is known that the Clostridia-rich microbiota can enhance the excretion of BAs in patients with diarrhea-predominant irritable bowel syndrome (Zhao et al., 2020). Additionally, DCA is a metabolite produced primarily by the genus *Clostridium* (Wang et al., 2020). The decreased abundance of Clostridia in IBD patients may finally result in reduced BA synthesis and excretion. Therefore, changes in the abundance of intestinal flora in IBD patients could decrease secondary BA levels.

We constructed a mouse model of DSS-induced enteritis and instilled DCA suspensions *via* enema (Fig. 3A). The combined treatment group showed greater weight loss (Fig. 3B), more obvious colonic mucosa edema (Fig. 3C), shorter colorectal length (Fig. 3D), more obvious inflammatory cell infiltration, more severe mucosal erosion (Fig. 3E), and a higher histopathological score (Fig. 3F) than the DSS group. These results confirm that DCA enema can aggravate enteritis in mice treated with DSS. In contrast, DCA intervention did not induce enteritis in healthy mice. Therefore, we hypothesized that, the increased excretion of bile acids exposed the inflamed gut to excess DCA, exacerbating enteritis. However, long-term hyperexcretion of DCA and the decreased abundance of Clostridia eventually leads to decreased blood and fecal DCA in patients with IBD.

BAs are known to affect cytokine levels. Chenodeoxycholic acid and ursodeoxycholic acid inhibit IL-1, IL-6, and TNF-*α* at certain concentrations by inhibiting the activity of monocytes (Calmus et al., 1992), while lithocholic acid downregulates NF-*κ*B activity *via* vitamin D receptors in colon cancer cells (Sun et al., 2008). In a high-fat diet-induced colitis mouse model, DCA promotes macrophage differentiation toward the M1 phenotype *via* Toll-like receptor 2 transduction *via* the M2 muscarinic receptor, leading to increased production of pro-inflammatory cytokines (Wang et al., 2020). In our study, RT-qPCR analysis of mouse colon tissue indicated that IL-1*β* was significantly highly expressed in the DCA treated DSS-induced group, but no difference was observed in expression of other cytokines. ELISA, western blotting, and immunohistochemical staining also showed an increase in IL-1*β* expression in the combined treatment group (Fig. 4). As a key pro-inflammatory cytokine, IL-1*β* is involved in various autoimmune inflammatory reactions and plays an important role in the pathogenesis, course, and severity of IBD (Nemetz et al., 1999; Pastorelli et al., 2013; Rubartelli et al., 1990). IL-1*β* is mainly produced by monocytes and macrophages and is also released from epithelial cells, endothelial cells, fibroblasts, synovial cells, neurons, mast cells, and glial cells. Flow cytometry analysis results indicated that the numbers of monocytes and macrophages in this study were not significantly different among the four groups. Therefore we hypothesize that the monocytes and macrophages may not contribute to the elevation in IL-1*β* expression, and the DCA-induced increase in IL-1*β* expression is likely attributed to epithelial or endothelial cells. However, the mechanism by which DCA regulates elevation in IL-1*β* expression requires further study.

Initial CD4^+^ T cells can differentiate into different subtypes of T cells under different conditions after antigenic stimulation. The types of cytokines and the balance between different cytokines play a key role in the regulation of helper T cell differentiation (Zhu, Yamane & Paul, 2010). UDCA is the first-line treatment for Primary biliary cholangitis (PBC). UDCA-treated nonresponders showed greater CD4^+^ T cell infiltration after treatment than before. Therefore, the degree of CD4^+^ T cell infiltration is also considered to be an indicator of PBC responds to UDCA treatment (Yu et al., 2021). In this study, in mouse intestinal LPMCs, the proportion of CD3^+^ T lymphocytes and CD4^+^ helper T lymphocytes increased in the combined treatment group (Figs. 5A and 5B), suggesting that DCA-induced aggravation of intestinal inflammation may be related, at least in part, to the activation of CD3^+^ and CD4^+^ T lymphocytes.

By analyzing the fecal flora of mice, we found that the relative abundance of Proteobacteria increased in the combined treatment group (Figs. 6A and 6B), consistent with the change observed in patients. Therefore, we believe that enteritis exacerbated by DCA in the mouse model also had an impact on the intestinal flora.

BAs bind to the TGR5 receptor on stem cells to promote their differentiation into tuft and goblet cells (Schneider, O’Leary & Locksley, 2019). Tuft cells located in the terminal ileum are an important component of the intestinal mucosa. In our study, the number of tuft cells was similar in the intestinal mucosa of IBD patients and healthy controls (Fig. 6C), possibly owing to the scarceness of tuft cells. However, tuft cells were observed in the small intestine of the mice, and the number of tuft cells in the combined treatment group was lower than that in the DSS group (Fig. 6D). Although tuft cells are rarely observed in the intestine, they play a key role in intestinal mucosal barrier formation. Tuft cells have been connected to group 2 innate lymphoid cells (ILC2s) in the small intestine in studies on parasites and protozoa over the last five years. Parasitic infections induce tuft cells to produce IL-25, followed by the activation of ILC2s to secrete IL-13, which in turn promotes epithelial crypt stem cells to differentiate into tuft cells. Consequently, an increased number of tuft cells contributes to the repair of the intestinal epithelium (Von Moltke et al., 2016). Our results suggest that DCA may reduce the number of tuft cells, thereby damaging the intestinal epithelium, weakening the intestinal mucosal barrier, and affecting immunity.

Intestinal DCA supplementation may weaken the intestinal mucosal barrier and aggravate intestinal inflammation by increasing the expression of IL-1*β*, activating CD3^+^ and CD4^+^ T cells, and reducing intestinal tuft cells. Therefore, BAs may affect intestinal inflammation in patients with IBD through intestinal immunity. Further research is needed to determine the specific mechanism by which DCA causes enteritis.

In another mouse model of colitis, feeding DCA induced intestinal inflammation which was associated with disturbed BA metabolism and gut microbiota (Xu et al., 2021). In contrast, DCA supplementation with the same concentration (5 mg DCA/150 ul water), also administered rectally, showed anti-inflammatory effects partially dependent on TGR5 signaling in mouse models of colitis (Sinha et al., 2020). Therefore, further studies are warranted to understand how to maintain the balance between the pro-inflammatory and anti-inflammatory effects of DCA. In the future, it may be possible to regulate intestinal inflammation in patients with IBD by changing the proportion of BAs in their diet. Moreover, since BA metabolism is dependent on the role of intestinal flora in patients, intestinal flora regulation is also an important part of the treatment of intestinal inflammation (Khoruts & Sadowsky, 2016). Further experiments are needed to verify whether changing the route of administration or the dose can produce therapeutic effects.

## Conclusions

Our study preliminarily explored the possible effect of the secondary BA DCA on intestinal inflammation in IBD patients and provided possible insights into why DCA aggravated intestinal inflammation in mice with DSS-induced enteritis.

It is believed that the reduction in fecal DCA level in IBD patients is related to disturbances in the intestinal flora. In this study, changes in the composition and abundance of gut microbiota led to lower serum and fecal secondary BA levels in IBD patients. This result indicates a correlation between the microbiota, BAs and their metabolites, and IBD.

Moreover, in this study, DCA increased the level of IL-1*β* while reducing the number of tuft cells and upregulating the expression of CD3^+^ and CD4^+^ T cells in the intestinal mucosa of mice with DSS-induced colitis, thereby affecting the intestinal mucosal barrier and intestinal immune functions and aggravating intestinal inflammation in the mouse model. Therefore, we believe that regulating intestinal BA levels may be of great significance in guiding the clinical diagnosis and treatment of patients with IBD. Although this study hypothesizes that the increase of IL1*β* induced by DCA may be derived from epithelial cells, the specific type of epithelial cells that secrete IL1*β* is not clear. In addition, changes in the number of tuft cells and the expression of CD3^+^ and CD4^+^ T cells are still superficial phenomena, and the specific mechanism is still unclear. In the future, it will be necessary to explore the specific mechanisms by which DCA affects intestinal inflammation and the relationship among DCA, tuft cells and immune system homeostasis.

##  Supplemental Information

10.7717/peerj.14842/supp-1 Figure S1 Pro-Inflammatory cytokines are elevated in IBD patients (A). The levels of IL-1 *β* have no significance within the groups. (B-C). IL-6 and IL-8 in active CD and UC were significantly higher than in the healthy control. IL-6 and IL-8 in active CD were significantly higher than in CD remission. (D)-(E). The levels of TNF *α*, IL-4, and IL-17A were not significantly different between IBD patients and healthy controls. (F). The IFN *γ* in active CD was significantly higher than that in healthy control.

10.7717/peerj.14842/supp-2 Table S1 Reagents and Laboratory Instruments

10.7717/peerj.14842/supp-3 Table S2 The criteria of histology analysis for colonic damage score

10.7717/peerj.14842/supp-4 Table S3 Primers for qRT-PCR

10.7717/peerj.14842/supp-5 Data S1 Raw data

10.7717/peerj.14842/supp-6 Supplemental Information 6 Beta-actin expression by western blot Beta-actin was the internal control (from right to left: water + water, water + DCA, DSS + water and DSS + DCA).

10.7717/peerj.14842/supp-7 Supplemental Information 7 Analysis of IL1beta expression by western blot Total protein was extracted from colon tissues of mice (water + water, water + DCA, DSS + water and DSS + DCA) and IL1beta antibody were used for detection (from left to right: water + water, water + DCA, DSS + water and DSS + DCA).

10.7717/peerj.14842/supp-8 Supplemental Information 8 Author Checklist

## Abbreviations

DSS: Dextran Sulfate Sodium
IBD: Inflammatory bowel disease
CD: Crohn’s disease
UC: Ulcerative colitis
TDCA: Taurodeoxycholic acid sodium
isoLCA: Isolithocholic acid
12-ketoLCA: 12-ketolithocholic acid
alloLCA: Allolithocholic acid
DCA: Deoxycholic acid
GLCA: Glycolithocholic acid
GCA: Glycocholic acid hydrate
UDCA: Ursodeoxycholic acid
TCA: Taurocholic acid
TCDCA: Taurochenodeoxycholic acid sodium salt
GUDCA: Glycoursodeoxycholic acid
CDCA: Chenodeoxycholic acid
CA: Cholic acid
TUDCA: Tauroursodeoxycholic acid Dihydrate
LCA: Lithocholic acid
GCDCA: Glycochenodeoxycholic acid sodium salt
TLCA: Taurolithocholic acid sodium salt
*α*-MCA: Alpha-Muricholic acid
GDCA: Glycodeoxycholic acid
IL: Interleukin
TNF*α*: tumor necrosis factor alpha
IFN*γ*: Interferon Gamma

## Additional Information and Declarations

### Data Availability

The following information was supplied regarding data availability: The raw measurements are available in the Supplementary Files.
