# Supplementary material for: Deoxycholic acid exacerbates intestinal inflammation by modulating interleukin-1β expression and tuft cell proportion in dextran sulfate sodium-induced murine colitis
Source: PeerJ. 2023 Feb 15;11:e14842. doi: 10.7717/peerj.14842 (PMC9938654; doi:10.7717/peerj.14842)
Supplement: Table S1 [file peerj-11-14842-s002.docx]

**Supplementary Table 1** list of the Reagents and Laboratory Instruments

| Deoxycholic acid | D2510, Sigma-Aldrich, St. Louis, MN |
| --- | --- |
| Dextran Sulfate Sodium | 0216011080, MP Biomedicalds, USA |
| pEGFR | ab40815, Abcam |
| Dcamkl1 | ab31704, Abcam |
| cyclooxygenase2 | sc-19999, SANTA CRUZ BIOTECHNOLOGY |
| EZ-press RNA Purification Kit | EZBioscience, USA |
| NanoVue spectrophotometer | GE Healthcare, Germany |
| PrimeScriptTM RT reagent Kit | Applied Biological Materials, Richmond, BC, Canada |
| GAPDH | Shenggong, Shanghai, China |
| RPMI 1640 medium | Thermo.C11875500BT, Invitrogen |
| RIPA lysis buffer | PC101, EpiZyme, Shanghai, China |
| protease inhibitor | GRF101, EpiZyme, Shanghai, China |
| BCA kit | P0012S, Beyotime, Shanghai, China |
| SDS-PAGE | P0015F, Beyotime, Shanghai, China |
| PVDF membrane (0.22μm) | Servicebio, Wuhan, China |
| interleukin-1β antibody | 26048-1-AP, Proteintech, Chicago, USA |
| β-actin antibody | 66009-1-Ig, Proteintech, Chicago, USA |
| TBST buffer | B548105, Sangon, shanghai, China |
| second antibody | SA00001-1, Proteintech, Chicago, USA |
| second antibody | SA00001-2, Proteintech, Chicago, USA |
| Enhanced Pico Light chemiluminescence Kit | SQ101, EpiZyme, Shanghai, China |
| Stool DNA Kit | Omega (EZNA), USA. |
| SYBR green | TaKaRa, Dalian, China |
| qRT-PCR platform | LightCycler 96, Roche, Switzerland |
| chloral hydrate | 30037516, Sinopharm, Shanghai, China |
| Enzyme linked immunosorbent assay (ELISA) reagent Kit (Murine IL-1β) | KE10003, Proteintech, Chicago, USA |
| ELISA reagent Kit (Human IL-1β) | KE00021, Proteintech, Chicago, USA |
| ELISA reagent Kit (Human IL-17A) | KE00203, Proteintech, Chicago, USA |
| ELISA reagent Kit (Human IFN-γ) | KE00146, Proteintech, Chicago, USA |
| ELISA reagent Kit (Human TNF-α) | KE00154, Proteintech, Chicago, USA |
| ELISA reagent Kit (Human IL-6) | KE00139, Proteintech, Chicago, USA |
| ELISA reagent Kit (Human IL-8) | KE00006, Proteintech, Chicago, USA |
| ELISA reagent Kit (Human IL-4) | KE00016, Proteintech, Chicago, USA |
| APC anti-mouse CD3 | 100236, BioLegend, California, USA |
| FITC anti-mouse CD4 | 100406, BioLegend, California, USA |
| PE anti-mouse CD8 | 100708, BioLegend, California, USA |
| PE/Cy7 anti-mouse B 220 | 552772, BD Biosciences, Franklin lakes, NJ, USA |
| APC anti-mouse CD11b | 17-0112, Invitrogen, USA |
| FITC anti-mouse F4/80 | 123108, BioLegend, California, USA |
| PerCP-cy5.5 anti-mouse Ly6C | 128012, BioLegend, California, USA |
| PE/Cy7 anti-mouse Ly6G | 552772, BD Biosciences, Franklin lakes, NJ, USA |
| PE anti-mouse CD11c | 25-5931, Invitrogen, USA |
| FITC anti-mouse MHC II | 107606, BioLegend, California, USA |
