# Supplementary material for: Deoxycholic acid exacerbates intestinal inflammation by modulating interleukin-1β expression and tuft cell proportion in dextran sulfate sodium-induced murine colitis
Source: PeerJ. 2023 Feb 15;11:e14842. doi: 10.7717/peerj.14842 (PMC9938654; doi:10.7717/peerj.14842)
Supplement: Table S2 [file peerj-11-14842-s003.docx]

**Supplementary Table 2** The criteria of histology analysis for colonic damage score

| Colon damage score | 0 | 1 | 2 | 3 |
| --- | --- | --- | --- | --- |
| Crypt architecture damage | None | Regeneration | Destruction |  |
| Edema in sub-mucosa | None | Mild | Moderate | Severe |
| Inflammatory cells infiltration | None/rare | Lamina propria | Sub-mucosa | Muscle layer |
