# Supplementary material for: Deoxycholic acid exacerbates intestinal inflammation by modulating interleukin-1β expression and tuft cell proportion in dextran sulfate sodium-induced murine colitis
Source: PeerJ. 2023 Feb 15;11:e14842. doi: 10.7717/peerj.14842 (PMC9938654; doi:10.7717/peerj.14842)
Supplement: Table S3 [file peerj-11-14842-s004.docx]

**Supplementary Table 3** Primers for qRT-PCR

| GAPDH-F | TGACCTCAACTACATGGTCTACA | IL8-F | TGTTGAGCATGAAAAGCCTCTAT |
| --- | --- | --- | --- |
| GAPDH-R | CTTCCCATTCTCGGCCTTG | IL8-R | AGGTCTCCCGAATTGGAAAGG |
| IL1β-F | GAAATGCCACCTTTTGACAGTG | IL17a-F | TCAGCGTGTCCAAACACTGAG |
| IL1β-R | TGGATGCTCTCATCAGGACAG | IL17a-R | CGCCAAGGGAGTTAAAGACTT |
| IL4-F | GGTCTCAACCCCCAGCTAGT | IFNγ-F | GCCACGGCACAGTCATTGA |
| IL4-R | GCCGATGATCTCTCTCAAGTGAT | IFNγ-R | TGCTGATGGCCTGATTGTCTT |
| IL6-F | CTGCAAGAGACTTCCATCCAG | TNFα-F | CAGGCGGTGCCTATGTCTC |
| IL6-R | AGTGGTATAGACAGGTCTGTTGG | TNFα-R | CGATCACCCCGAAGTTCAGTAG |
