# Supplementary material for: Deoxycholic acid exacerbates intestinal inflammation by modulating interleukin-1β expression and tuft cell proportion in dextran sulfate sodium-induced murine colitis
Source: PeerJ. 2023 Feb 15;11:e14842. doi: 10.7717/peerj.14842 (PMC9938654; doi:10.7717/peerj.14842)
Supplement: Data S1 [file peerj-11-14842-s005.pdf]

Figure1

Group	CA	DCA	CDCA	UDCA	LCA	GCA	GDCA
A-CD	3.03	0.795	23.4	0	0	74.8	0
A-CD	3.98	419	104	27.4	25.6	86.9	182
A-CD	4.18	5.22	63.9	4.32	0.8	86.8	0
A-CD	4.34	2090	124	341	84.9	6.31	45.9
A-CD	4.4	0	54.2	1.89	0	30.8	0
A-CD	5.94	4.77	13.8	3.49	0.269	248	0
A-CD	6.08	62.3	77.4	86.3	0	7.05	33
A-CD	6.88	1.47	46	3.95	0	99.5	0
A-CD	7.5	2	285	31.7	0	134	0
A-CD	7.93	104	40.7	0	5.04	168	219
A-CD	8.75	141	34.8	35.7	4.6	96.6	45.5
A-CD	8.8	0.738	20.2	0	0	2190	0
A-CD	10.5	5.78	18.7	0	0	74.3	0
A-CD	11.4	0	101	137	0	25	0
A-CD	12.5	0	43.1	0.58	0	60.8	0
A-CD	13.2	0.39	59.6	47.1	3.05	242	0
A-CD	13.7	0	127	25	0	188	0
A-CD	13.8	44.5	150	12.8	0	274	248
A-CD	16	1.33	110	3.45	0	280	0
A-CD	17.6	1.91	527	110	0	18	0
A-CD	17.8	88.7	34.9	11.5	0	65.7	32.9
A-CD	17.9	144	119	38.4	18.8	90.3	109
A-CD	18.2	0	154	34.2	2.49	66.6	0
A-CD	18.5	0	16.1	5.68	0	220	0
A-CD	18.7	62	29.9	39.4	4.04	219	111
A-CD	20.5	155	20	31.5	1.62	88.2	107
A-CD	22	0	28.4	0	0	245	0
A-CD	23.4	0.486	149	0	0.756	137	0
A-CD	23.7	30.8	151	77.8	0	135	14.2
A-CD	23.9	124	85	13.7	4.07	53.7	42.8
A-CD	27.9	1.26	133	58	0	206	0
A-CD	28.1	45.3	384	358	0	45.3	0
A-CD	30.1	3.42	136	0	0.75	122	0
A-CD	31.2	1.33	155	139	0	86.7	0
A-CD	31.6	5.03	129	32.2	13.1	476	0
A-CD	35.2	2.94	507	1.33	0	144	0
A-CD	36.2	8.96	797	3.76	0	241	0
A-CD	36.5	1.2	302	5.78	0	261	2.68
A-CD	37.4	2590	37.1	47.3	15	0	51.7
A-CD	40.1	14	314	4.85	0	184	0
A-CD	45.3	3.25	197	206	1.82	153	0
A-CD	48.7	2.37	477	587	1.84	19.1	0
A-CD	49.3	295	347	67	0.144	54.9	18.9
A-CD	70	0	162	1.06	3.59	223	0.91
A-CD	79.1	27.1	347	444	0	125	22.4
A-CD	84.8	0	89.4	38.4	0	2720	0
A-CD	86	17.1	376	17.9	0	370	0
A-CD	114	0	550	0.12	0	498	0
A-CD	132	0	211	25.8	0	105	0
A-CD	151	0	1190	80.2	0	50.8	1.15
A-CD	154	3.8	535	23.5	0	559	0
A-CD	155	0	2880	41.2	0	95.9	0
A-CD	168	0	307	2.26	0	885	0
A-CD	198	204	544	46	5.02	232	167
A-CD	199	0.573	387	0	0	4950	0
A-CD	225	0	871	0	0	568	0

A-CD	234	2.89	20.1	0	0	291	8.45
A-CD	245	2.25	564	49	0	207	0
A-CD	367	55.1	2910	842	0	927	3.18
A-CD	391	0	471	0	0	370	0.06
A-CD	464	0.09	1060	44.6	0	146	2.96
A-CD	578	4.37	6790	198	0	67.4	0
A-CD	620	756	2600	121	3.27	174	96.3
A-CD	621	0	1430	16.8	2.48	131	0
A-CD	1130	560	2150	82.7	2.42	107	89
A-CD	4580	4860	16600	54.6	0	135	299
Re-CD	2.3	74.6	3.25	4.74	0	13.1	5.67
Re-CD	2.37	2.86	10.4	0	0.317	2770	0
Re-CD	3.95	8.86	0	0	4.56	3770	0
Re-CD	4.5	0	47.4	0	0	65.2	0
Re-CD	6.43	0.937	67.2	0	0	454	0
Re-CD	7.56	74.1	92.5	20.9	0	258	195
Re-CD	7.61	65.8	117	249	14.2	20.3	0
Re-CD	9.2	865	48.6	240	31.2	15.4	119
Re-CD	9.31	9.58	110	3.36	0	57.3	14.5
Re-CD	9.44	11.9	47.3	10.9	0	302	0
Re-CD	11.6	0	56	34.3	0	80.6	0
Re-CD	12.7	0	70.8	7.83	0	236	0
Re-CD	16.1	28.5	58.6	9.05	3.68	125	102
Re-CD	16.7	0	39.5	21.8	0	28.2	0
Re-CD	17.2	348	212	150	25	470	762
Re-CD	17.8	480	249	78.1	42.4	73.6	437
Re-CD	19.2	20.4	493	40.1	0.18	207	18.4
Re-CD	20.1	2.73	45.6	2.74	0	258	0
Re-CD	20.7	9.72	229	34.2	0	63.3	0
Re-CD	22.6	2.23	107	5.56	0	361	0
Re-CD	23.7	0	93.7	1.62	0	249	4.82
Re-CD	25.1	0.227	25.1	6.74	0	385	0
Re-CD	25.9	2.23	1360	56.1	0	90.5	0
Re-CD	29.8	3.41	174	0	0	36.6	0
Re-CD	29.8	2.26	31.5	0	0	41.9	0
Re-CD	41.1	1.24	758	27.3	0	49.6	0
Re-CD	48	163	123	34	2.61	245	101
Re-CD	49.9	2.78	538	236	0	224	2.35
Re-CD	53.8	153	345	27.9	0	101	110
Re-CD	63.5	0	587	2.53	0	249	0
Re-CD	63.6	0.82	658	177	13.6	15.9	0
Re-CD	64.4	16.1	132	76.2	0	175	0
Re-CD	64.9	7.03	411	73	2.22	73.2	0
Re-CD	77	150	383	201	0	90.9	61.7
Re-CD	98.9	0.24	141	0	0.48	489	0
Re-CD	99.7	0	1190	924	0	258	0
Re-CD	106	13.3	806	95.1	0	134	1.76
Re-CD	115	1.73	778	314	0.362	126	0
Re-CD	119	6.36	2790	364	0	90	0
Re-CD	149	148	224	71	0	28.2	47.8
Re-CD	149	6.44	1100	107	1.77	348	0
Re-CD	160	3.1	404	37.2	0	105	0
Re-CD	165	3.96	743	66.8	0	80.2	0
Re-CD	191	5.2	1510	701	0	71.1	0
Re-CD	213	91.9	855	166	0	203	95.9
Re-CD	269	12.1	2180	69.1	0	94.1	0
Re-CD	308	10.9	1080	58.5	0	38.2	0
Re-CD	374	15.7	7420	3740	0	93.8	0

Re-CD	404	1370	1040	214	7.77	46.2	188
Re-CD	430	11.1	2080	662	0	487	3.35
Re-CD	481	605	603	47.1	0.9	44.3	80.6
Re-CD	562	12.4	3120	268	0	606	0
Re-CD	649	315	3900	134	0	889	289
Re-CD	719	0	1990	839	0	75.4	0
Re-CD	802	7.33	5030	262	0	19.1	0
Re-CD	849	34.1	2980	353	0	98.8	0.00796
Re-CD	1020	32.7	3500	764	0	188	8.73
Re-CD	3110	0	5120	661	1.21	2460	0
Re-CD	5000	0	17500	456	0	153	0
Re-CD	7430	7210	8240	1020	0.54	912	879
A-UC	9.14	14.9	82.6	2.43	0	290	4.57
A-UC	13.4	3.44	26.1	20.9	0	108	23
A-UC	13.6	37.5	56.5	7.35	0	82	66.5
A-UC	17.2	0	11.1	5.98	0	52.2	4.46
A-UC	19.6	36.4	39.3	19.9	1.38	133	57.9
A-UC	19.8	401	64.6	48.7	3.13	103	75.8
A-UC	20	39.9	39.9	20	29.9	30.1	10
A-UC	20.2	12.7	51.2	11.2	0	439	12.1
A-UC	22.8	50.7	132	11.5	0	251	11.4
A-UC	30	61.4	159	42.6	0	77.4	17.2
A-UC	30.5	141	193	88.6	4.96	117	51.3
A-UC	30.5	202	238	30.9	2.38	332	190
A-UC	31.9	0.244	17.1	0	4.36	365	1.31
A-UC	34.1	14.3	30.1	0	0.166	220	44
A-UC	36	8.81	47.4	3.47	0	116	26
A-UC	41.1	35.6	1140	45.7	3.08	785	137
A-UC	65	3.44	78.9	118	0	324	0
A-UC	122	16.5	322	47.7	0	80.5	0
A-UC	132	10.5	851	467	0	97.3	0
A-UC	142	1.16	208	9.43	0	285	0
A-UC	221	10.9	744	5.75	6.34	282	0
A-UC	277	8.46	4660	892	0	529	0
A-UC	326	0	672	254	0	217	0
A-UC	1310	12.3	2240	20.4	0.059	314	0
A-UC	1750	59.9	1790	13.7	0	731	19.7
A-UC	4320	2.8	5460	326	0	324	0
Re-UC	5.18	38	22.9	4.41	0	23.2	11.6
Re-UC	5.32	0	33.3	0	0	22.3	0
Re-UC	22.3	109	419	258	0	28.6	19.1
Re-UC	81.9	31.6	331	69.5	1.61	71.9	15.5
Re-UC	882	635	1620	174	0	165	178
HC	7.35	8.02	40	8.61	0	38.3	13.3
HC	8.18	0	84.2	4.21	0	67.2	0
HC	8.99	287	139	85.6	14.3	122	101
HC	10.5	81.8	39	8.22	0	11.2	26.2
HC	12.7	36.6	39.5	8.17	0	30.7	8.98
HC	13.9	41.2	195	43.4	9.06	64.6	24.6
HC	16.3	16.5	27	3.48	0	138	0
HC	16.9	11.9	39	2.14	0	33	0
HC	17.4	369	203	193	19.2	77.1	80.5
HC	18.2	288	43.3	111	26.3	140	338
HC	18.4	26.7	272	109	2.46	98.7	0
HC	19.4	135	90	13.5	43.2	42.1	61.5
HC	19.5	41.9	41.5	5.43	3.7	138	89.3
HC	20.4	72.9	68	11.4	4.08	97.1	70.7
HC	20.5	26.3	93.1	5.95	0	1430	0

HC	21.6	223	90.4	21.8	15	297	171
HC	22.3	10.2	86.8	20	0	72.7	8.66
HC	22.5	104	90.6	18.1	2.27	64.2	25.2
HC	24.1	683	84.5	61.7	15.7	40.7	72
HC	25.2	16.7	308	39.2	1.36	163	9.18
HC	27.3	5.24	609	12.5	13	280	0
HC	27.8	394	423	71.1	34.5	431	378
HC	30.1	1.9	87.9	4.16	0	315	15.5
HC	31.7	271	168	48	4.98	13.7	10.1
HC	34.1	521	107	58.7	2.13	47.3	117
HC	34.3	676	101	97.1	35.1	73.3	61
HC	34.9	6.35	181	177	0	58.4	2.66
HC	35.4	134	367	57.2	7.45	67.3	84.2
HC	39.1	32.6	485	85.1	0	54	0
HC	39.4	12.8	172	5.46	6.81	41.3	3.89
HC	43.9	1080	423	203	33	54	54.8
HC	59.9	155	819	121	0	246	127
HC	61.9	3.23	558	6.33	0	156	0
HC	74.2	185	293	61.2	4.5	233	312
HC	75.9	8.04	212	21	0	556	0
HC	77.5	17	581	197	0	1510	37
HC	84.1	248	253	76.1	2.33	153	97.7
HC	90.2	17.7	1170	157	0	270	0
HC	105	121	171	16.6	2.82	120	42.1
HC	108	16.5	329	81.2	0	122	0
HC	119	7.37	453	453	0	586	1.42
HC	120	93.8	847	250	0	180	13
HC	128	519	394	111	2.94	611	593
HC	136	6.79	302	62.3	1.9	369	0
HC	138	16.5	180	18.8	16	88	27.4
HC	139	29.4	1060	139	0	204	5.59
HC	140	51.9	1010	211	0.772	298	19.4
HC	154	19.5	787	45.8	0	67.5	0
HC	169	173	2190	347	0	72.1	23.4
HC	177	116	1750	6.36	0	148	53.9
HC	180	791	663	83.8	4.98	87.1	259
HC	232	1.56	1390	33.1	0	118	0
HC	241	142	1800	527	2.11	115	31.9
HC	277	307	1520	635	0.906	971	469
HC	514	923	15400	1220	0	120	39.9
HC	566	1040	869	119	21.6	101	279
HC	2310	880	5200	530	0	242	90.8
HC	2650	963	1090	108	6.05	77	74.8

Figure2
A and B

Component	CD	CD	CD	CD	CD	HC	HC
12-ketolithochol	0	0	0	0	0	14704.19	96178.92
23-Norcholic aci	105.851612	372.5383902	20.678134	131.35412	33.09572	132.2494	348.0322
3-dehydrocholic	14657.4976	13208.89011	220.83987	1369.1451	0	5412.403	1895.466
3β -Ursodeoxych	49736.9493	88415.96806	0	0	0	19823.12	13548.4
7-Ketolithocholi	175829.087	78465.09	396.38665	13379.963	0	13153.51	7972.099
Allolithocholic ac	0	108.1474723	0	0	0	814.5569	2252.324
Alpha-Muricholi	153819.093	213235.4712	3455.2089	14531.758	0	103698.3	89501.02
Chenodeoxycho	172328.815	77942.81188	0	199421.7	0	162911.9	108536.9
Chenodeoxycho	0	0	0	11422.049	381.82	123.5983	287.1699
Cholic acid	311814.424	401673.7442	8795.468	384355.22	393.9938	202371	139263.4
Deoxycholic acic	44350.0442	871.5328809	0	3119.211	0	156282.8	1091439

Glycochenodeox	1769.99503	4702.270701	0	17495.73	72321.29	1105.701	701.9937
Glycocholic acid	2259.10739	1777.469675	217.10911	27495.156	41965.25	200.0936	229.865
Glycodeoxycholi	113.715199	0	0	186.25633	0	90.37489	113.7885
Glycohyocholic a	608.691641	1250.746366	0	0	0	367.9936	319.9629
Glycolithocholic	0	0	0	0	0	0	0
Glycoursodeoxy	218.465249	1777.046865	71.668759	9679.1922	1373.574	87.42909	36.4058
Hyodeoxycholic	187098.386	304417.9456	1398.3987	90388.805	0	81424.67	44961.16
Isolithocholic aci	0	58.92062998	0	0	0	684.5562	1412.2
Tauro-alpha-Mu	1128.09798	306.7974228	0	0	276.0647	0	22.52876
Taurochenodeox	905.144176	553.4076345	0	2134.4239	38908.02	70.99259	114.0643
Taurocholic acid	4528.428	4750.23218	0	2660.9613	37414.11	258.127	101.8991
Taurodeoxycholi	190.694169	0	0	0	0	32.26353	352.9418
Taurohyocholic a	940.845122	0	0	567.37588	0	0	0
Taurohyodeoxyc	410.892833	4088.822685	0	1951.4732	574.2682	50.21152	38.64357
Taurolithocholic	0	36.09320752	0	0	0	0	25.67098
Tauroursodeoxy	503.425104	4758.808358	0	2477.5937	644.3714	44.34081	31.80032
Ursodeoxycholic	183459.774	309476.6297	1651.7902	87111.641	0	81995.81	44678.95
lithocholic acid	3054.94809	5411.161354	1345.8104	312.12371	0	180882	1920430
lithocholic acid 3	2232.01337	669.4268549	41.768811	2110.5652	26.5142	412.4944	104269.7

A and B

Component	CD	CD	CD	CD	CD	UC	UC
Glycoursodeoxy	218.465249	1777.046865	71.668759	9679.1922	1373.574	35.50731	0
Tauroursodeoxy	503.425104	4758.808358	0	2477.5937	644.3714	251.8101	0
Alpha-Muricholi	153819.093	213235.4712	3455.2089	14531.758	0	33804.45	0
Taurohyodeoxyc	410.892833	4088.822685	0	1951.4732	574.2682	291.8574	0
Hyodeoxycholic	187098.386	304417.9456	1398.3987	90388.805	0	523.5072	524.4229
Ursodeoxycholic	183459.774	309476.6297	1651.7902	87111.641	0	570.141	631.0626
lithocholic acid	3054.94809	5411.161354	1345.8104	312.12371	0	1107.316	309.3004
Chenodeoxycho	0	0	0	11422.049	381.82	477.7987	1157.559
Glycochenodeox	1769.99503	4702.270701	0	17495.73	72321.29	2188.936	950.5341
Deoxycholic acid	44350.0442	871.5328809	0	3119.211	0	0	0
Glycocholic acid	2259.10739	1777.469675	217.10911	27495.156	41965.25	1896.958	1410.624
3β-Ursodeoxych	49736.9493	88415.96806	0	0	0	0	0
Tauro-alpha-Mu	1128.09798	306.7974228	0	0	276.0647	686.3799	0
Allolithocholic ac	0	108.1474723	0	0	0	0	0
Glycolithocholic	0	0	0	0	0	0	0
Isolithocholic aci	0	58.92062998	0	0	0	0	0
Taurolithocholic	0	36.09320752	0	0	0	0	0
hyocholic acid	0	0	0	0	0	0	0
3-dehydrocholic	14657.4976	13208.89011	220.83987	1369.1451	0	4046.724	525.9642
23-Norcholic aci	105.851612	372.5383902	20.678134	131.35412	33.09572	84.39245	239.3216
Glycodeoxycholi	113.715199	0	0	186.25633	0	0	0
Taurochenodeox	905.144176	553.4076345	0	2134.4239	38908.02	14728.46	318.2585
Cholic acid	311814.424	401673.7442	8795.468	384355.22	393.9938	111193.1	246087.1
lithocholic acid 3	2232.01337	669.4268549	41.768811	2110.5652	26.5142	346.4057	2268.315
Chenodeoxycho	172328.815	77942.81188	0	199421.7	0	63113	15112.36
7-Ketolithocholi	175829.087	78465.09	396.38665	13379.963	0	33631.08	22719.48
Taurodeoxycholi	190.694169	0	0	0	0	0	0
Taurocholic acid	4528.428	4750.23218	0	2660.9613	37414.11	9117.967	4285.885
Glycohyocholic a	608.691641	1250.746366	0	0	0	0	395.7623
Taurohyocholic a	940.845122	0	0	567.37588	0	756.4199	0

C

OTU ID	HC	HC	CD	CD	CD	UC	UC
Actinobacteriota	0.06456371	0.090548054	0.0204244	0.1719051	0.007489	0.033587	0.002269
Bacteroidota	0.00896403	0.021332123	0.2444117	0.0224668	0.721207	0.000681	0.835697
Cyanobacteria	0	0	0.003631	0	0	0.019744	0
Firmicutes	0.86769545	0.855667764	0.4671508	0.7754454	0.068989	0.221037	0.120958
Proteobacteria	0.05026665	0.032452059	0.2643822	0.0301827	0.202315	0.724952	0.041076

Unclassified	0	0	0	0	0	0	0
Verrucomicrobic	0.00851016	0	0	0	0	0	0

D

	HC1	HC2	CD1	CD2	UC1	UC2	UC3
OTU ID	FC	JJY	WYX	YYB	YCS	YT	QBL
Actinobacteriota	0.06456371	0.090548054	0.0204244	0.1719051	0.033587	0.002269	0.201634
Bacteroidota	0.00896403	0.021332123	0.2444117	0.0224668	0.000681	0.835697	0.09259
Cyanobacteria	0	0	0.003631	0	0.019744	0	0
Firmicutes	0.86769545	0.855667764	0.4671508	0.7754454	0.221037	0.120958	0.685351
Proteobacteria	0.05026665	0.032452059	0.2643822	0.0301827	0.724952	0.041076	0.018722
Unclassified	0	0	0	0	0	0	0.001702
Verrucomicrobic	0.00851016	0	0	0	0	0	0

Component	FC	JJY	WYX	YYB	YCS	YT	QBL
12-ketolithochol	96178.9194	115543.6675	0	0	0	0	0
Allolithocholic ac	2252.32424	13201.9411	0	108.14747	0	0	0
Alpha-Muricholi	89501.0169	52627.31986	153819.09	213235.47	33804.45	0	663.0265
Deoxycholic acic	1091438.71	476077.2702	44350.044	871.53288	0	0	3551.927
Isolithocholic aci	1412.2002	14202.11607	0	58.92063	0	0	0
Taurodeoxycholi	352.941789	2634.298707	190.69417	0	0	0	292.1307
lithocholic acid	1920430.41	1735222.134	3054.9481	5411.1614	1107.316	309.3004	0

E

OTU ID	FC	JJY	WYX	YYB	QBL	YCS	YT
Actinobacteriota	0.06456371	0.090548054	0.0204244	0.1719051	0.201634	0.033587	0.002269
Bacteroidota	0.00896403	0.021332123	0.2444117	0.0224668	0.09259	0.000681	0.835697
Cyanobacteria	0	0	0.003631	0	0	0.019744	0
Firmicutes	0.86769545	0.855667764	0.4671508	0.7754454	0.685351	0.221037	0.120958
Proteobacteria	0.05026665	0.032452059	0.2643822	0.0301827	0.018722	0.724952	0.041076
Unclassified	0	0	0	0	0.001702	0	0
Verrucomicrobic	0.00851016	0	0	0	0	0	0

E

OTU ID	FC	JJY	WYX	YYB	QBL	YCS	YT
Actinobacteria	0.04992625	0.089640304	0.0047657	0.1719051	0.200045	0.030296	0.002156
Alphaproteobact	0.01214116	0.004084875	0.0498128	0.0179281	0.006468	0.337796	0.016566
Bacilli	0.02655169	0.005673437	0.0851016	0.733235	0.327811	0.216044	0.096221
Bacteroidia	0.00896403	0.021332123	0.2444117	0.0224668	0.09259	0.000681	0.835697
Clostridia	0.82366958	0.849994327	0.2787927	0.0415296	0.356065	0.004993	0.018722
Coriobacteriia	0.014524	0.00090775	0.0154317	0	0.001589	0	0
Cyanobacteriia	0	0	0.003631	0	0	0.019744	0
Gammaproteoba	0.0381255	0.028367185	0.2145694	0.0122546	0.012255	0.387155	0.024509
Negativicutes	0.01747419	0	0.1032566	0.0006808	0.001475	0	0.006014
Rubrobacteria	0.00011347	0	0.0002269	0	0	0.003291	0.000113
Unclassified	0	0	0	0	0.001702	0	0
Verrucomicrobia	0.00851016	0	0	0	0	0	0

OTU ID	FC	JJY	WYX	YYB	QBL	YCS	YT
Bacteria	1	1	1	1	0.998298	1	1
Unclassified	0	0	0	0	0.001702	0	0

OTU ID	FC	JJY	WYX	YYB	QBL	YCS	YT
Acidaminococca	0	0	0.0487916	0	0	0	0
Akkermansiacea	0.00851016	0	0	0	0	0	0
Alicyclobacillace	0	0	0	0	0	0.008851	0
Atopobiaceae	0	0	0	0	0.000227	0	0
Bacillaceae	0.00011347	0	0	0	0.000454	0.000681	0.000227
Bacteroidaceae	0.00453875	0.020651311	0.2427096	0.0163395	0.082378	0.000681	0.816635
Beijerinckiaceae	0.00022694	0.000226937	0	0.0026098	0.000681	0	0.001929
Bifidobacteriacea	0.0493589	0.088959492	0	0.1662317	0.198457	0.001816	0

Burkholderiacea	0.0203109	0.001588562	0.059798	0.0029502	0.004766	0.382049	0.012255
Butyricicoccacea	0	0	0	0	0.002269	0	0
Carnobacteriace	0.00022694	0.000226937	0	0.0037445	0.000227	0	0.003177
Caulobacteracea	0.00102122	0	0.0049926	0.0001135	0.00034	0.062975	0
Chitinophagacea	0.00442528	0.000680812	0.001702	0.0061273	0.003971	0	0.019063
Chloroplastnorar	0	0	0.003631	0	0	0.019744	0
ClostridiaUCG01	0	0	0	0	0.000227	0	0
Clostridiaceae	0	0	0	0.0182685	0	0	0
Comamonadace	0.00011347	0	0	0	0	0.004766	0
Coriobacteriacea	0.014524	0	0	0	0	0	0
Corynebacteriac	0	0	0	0	0.000113	0	0
Eggerthellaceae	0	0.00090775	0.0154317	0	0.001362	0	0
Enterobacteriace	0.00283672	0.025757404	0.1241348	0.0004539	0.000227	0	0.009531
Enterococcaceae	0	0	0.0044253	0.0144105	0.006922	0	0
Erysipelatoclostri	0	0.001928969	0.010893	0.0014751	0.000567	0	0
Erysipelotrichace	0	0	0.0021559	0	0.000454	0	0
Eubacteriaceae	0	0	0	0	0.025417	0	0
Hydrogenophila	0	0	0	0	0	0.00034	0.002269
Labraceae	0	0	0	0.0002269	0.000227	0	0.000227
Lachnospiraceae	0.22705095	0.781232271	0.2210371	0.0180415	0.167593	0.001362	0
Lactobacillaceae	0	0	0.0057869	0.509815	0.312493	0	0.002156
Microbacteriacea	0.00011347	0.000680812	0.0040849	0.005333	0.000908	0	0.000681
Micrococcaceae	0.00011347	0	0	0	0	0.026438	0
Monoglobaceae	0.00295019	0	0	0	0	0	0
Mycobacteriacea	0.00011347	0	0	0.0003404	0.00034	0	0.001475
Oscillospiraceae	0.00329059	0.000567344	0.0009078	0	0.155679	0	0
Peptostreptococ	0.02121865	0.010666062	0.0169068	0.0052196	0.000113	0	0.018722
Propionibacteria	0.00022694	0	0.0006808	0	0.000227	0.002042	0
Pseudomonadac	0.00068081	0.000113469	0	0.0007943	0	0	0
Reyranellaceae	0	0	0	0	0.000454	0	0
Rhizobiaceae	0.00249631	0.00272325	0.005333	0.0114603	0.001248	0.000113	0.007716
Rhodanobactera	0.00703506	0.000113469	0.0031771	0.0066947	0.003404	0	0.000454
Rubrobacteriace	0.00011347	0	0.0002269	0	0	0.003291	0.000113
Ruminococcacea	0.56337229	0.057074776	0.0312039	0	0.004766	0.003631	0
Sphingomonada	0.00714853	0.000794281	0.0369908	0.0007943	0.001475	0.270056	0.001362
Sporolactobacilla	0.01214116	0.001361625	0.021786	0.0011347	0.001816	0.205378	0.009872
Staphylococcace	0	0	0	0	0.000227	0.000681	0
Streptococcacea	0.01407012	0.002155906	0.0400545	0.2026552	0.004652	0.000454	0.08079
Sutterellaceae	0	0	0	0	0.00034	0	0
Tannerellaceae	0	0	0	0	0.006241	0	0
Unclassified	0	0	0	0	0.001702	0	0
Veillonellaceae	0.01747419	0	0.054465	0.0006808	0.001475	0	0.006014
Xanthobacterace	0.00124816	0.000340406	0.0024963	0.0027233	0.002042	0.004652	0.005333
Xanthomonadac	0.00714853	0.000794281	0.0274594	0.0013616	0.003518	0	0
[Eubacterium] cc	0.00578691	0.000453875	0.0087371	0	0	0	0

OTU ID	FC	JJY	WYX	YYB	QBL	YCS	YT
Aeriscardovia	0	0	0	0	0.178713	0	0
Agathobacter	0.00204244	0.013162374	0	0	0	0	0
Akkermansia	0.00851016	0	0	0	0	0	0
Anaerostipes	0.00102122	0.154657892	0.008964	0	0	0.000454	0
Asinibacterium	0.00045388	0	0.0002269	0.0001135	0	0	0.000113
Bacillus	0.00011347	0	0	0	0.000454	0.000681	0.000227
Bacteroides	0.00453875	0.020651311	0.2427096	0.0163395	0.082378	0.000681	0.816635
Bifidobacteriacea	0	0	0	0	0.000227	0	0
Bifidobacterium	0.0493589	0.088959492	0	0.1662317	0.019517	0.001816	0
Blautia	0.07035062	0.187790764	0.1071145	0	0.055827	0	0
Bradyrhizobium	0.00056734	0.000340406	0.0021559	0.0022694	0.001135	0.004539	0.004993

BurkholderiaCab	0.00034041	0	0	0.0006808	0.000794	0	0
Butyricicoccus	0	0	0	0	0.002269	0	0
Caulobacter	0.00090775	0	0.0049926	0.0001135	0.00034	0.032112	0
Caulobacteracea	0.00011347	0	0	0	0	0.030863	0
Chitinophaga	0.00079428	0	0.0009078	0.0020424	0.000567	0	0.003971
Chloroplastnorai	0	0	0.003631	0	0	0.019744	0
ClostridiaUCG01	0	0	0	0	0.000227	0	0
Clostridiumsensu	0	0	0	0.0182685	0	0	0
Collinsella	0.014524	0	0	0	0	0	0
Comamonas	0.00011347	0	0	0	0	0	0
Cutibacterium	0.00022694	0	0.0006808	0	0.000227	0.002042	0
Delftia	0	0	0	0	0	0.004766	0
Dialister	0.01747419	0	0.0120277	0.0002269	0	0	0
Dorea	0.01214116	0.057074776	0	0	0	0	0
Eggerthella	0	0.00090775	0.0154317	0	0.001362	0	0
Enterobacter	0	0	0.0006808	0	0	0	0.002042
Enterococcus	0	0	0.0044253	0.0144105	0.006922	0	0
Erysipelatoclostri	0	0	0	0	0.000227	0	0
Erysipelatoclostri	0	0.001928969	0.010893	0.0014751	0.00034	0	0
EscherichiaShige	0.00283672	0.025757404	0.123454	0.0004539	0.000227	0	0.006354
Eubacterium	0	0	0	0	0.025417	0	0
Faecalibacterium	0.21411551	0.026665154	0.0293884	0	0	0.002837	0
Flavonifractor	0	0.000567344	0.0009078	0	0.155679	0	0
Fusicatenibacter	0.06966981	0.187904232	0	0	0	0	0
Granulicatella	0.00022694	0.000226937	0	0.0037445	0.000227	0	0.003177
Howardella	0	0	0	0.0080563	0	0	0
Hungatella	0	0.001248156	0	0	0.003291	0.00034	0
Hydrogenophilu	0	0	0	0	0	0.00034	0.002269
IncertaeSedis	0	0.00090775	0.0009078	0	0.004766	0	0
Intestinibacter	0.00317713	0.006127312	0.0007943	0.0032906	0	0	0
Klebsiella	0	0	0	0	0	0	0
Labrys	0	0	0	0.0002269	0.000227	0	0.000227
Lachnoclostridiu	0.0018155	0.001134687	0.0062408	0.0047657	0.004425	0	0
Lachnospira	0.00022694	0	0	0	0.000454	0	0
Lachnospiraceae	0.00011347	0.000226937	0	0	0	0	0
Lachnospiraceae	0	0.005446499	0.0263247	0	0.011687	0	0
Lacticaseibacillus	0	0	0	0.0012482	0	0	0
Lactobacillus	0	0	0.0002269	0.0002269	0.183025	0	0.001816
Lawsonella	0	0	0	0	0.000113	0	0
Ligilactobacillus	0	0	0.0054465	0.4925678	0.117213	0	0.00034
Limosilactobacill	0	0	0.0001135	0.0157722	0.00295	0	0
Mesorhizobium	0.00079428	0.000567344	0.0023828	0.0041983	0.001021	0.000113	0.003291
Methylovirgula	0.00022694	0.000226937	0	0.0026098	0.000681	0	0.001929
Microbacterium	0.00011347	0.000680812	0.0040849	0.005333	0.000908	0	0.000681
Monoglobus	0.00295019	0	0	0	0	0	0
Mycobacterium	0.00011347	0	0	0.0003404	0.00034	0	0.001475
Olsenella	0	0	0	0	0.000227	0	0
Parabacteroides	0	0	0	0	0.006241	0	0
Pediococcus	0	0	0	0	0.009304	0	0
Peptostreptococ	0	0.000113469	0.0040849	0.0001135	0.000113	0	0.018722
Phascolarctobac	0	0	0.0487916	0	0	0	0
Phyllobacterium	0.00170203	0.002155906	0.0029502	0.007262	0.000227	0	0.004425
Pseudarthrobact	0	0	0	0	0	0.014637	0
Pseudolabrys	0	0	0	0	0.000227	0	0
Pseudomonas	0.00068081	0.000113469	0	0.0007943	0	0	0
Ralstonia	0.0199705	0.001588562	0.059798	0.0022694	0.003971	0.382049	0.012255
Renibacterium	0.00011347	0	0	0	0	0.011801	0
Reyranella	0	0	0	0	0.000454	0	0

Rhodanobacter	0.00703506	0.000113469	0.0031771	0.0066947	0.003404	0	0.000454
Rhodopseudomo	0.00056734	0	0	0	0.000227	0.000113	0.000227
Romboutsia	0.01804153	0.004425281	0.0120277	0.0018155	0	0	0
Roseburia	0.0417565	0	0	0	0	0.000227	0
Rubrobacter	0.00011347	0	0.0002269	0	0	0.003291	0.000113
Ruminococcus	0.0417565	0	0.0009078	0	0	0.000227	0
Salmonella	0	0	0	0	0	0	0.001135
Sellimonas	0	0	0	0	0.027346	0	0
Sphingomonas	0.00714853	0.000794281	0.0369908	0.0007943	0.001475	0.270056	0.001362
Sporolactobacill	0.01214116	0.001361625	0.021786	0.0011347	0.001816	0.205378	0.009872
Staphylococcus	0	0	0	0	0.000227	0.000681	0
Stenotrophomor	0.00714853	0.000794281	0.0274594	0.0013616	0.003518	0	0
Streptococcus	0.01407012	0.002155906	0.0400545	0.2026552	0.004652	0.000454	0.08079
Subdoligranulum	0.30750028	0.029501872	0	0	0	0.000567	0
Sutterella	0	0	0	0	0.00034	0	0
Tumebacillus	0	0	0	0	0	0.008851	0
Tyzzerella	0	0.000680812	0	0	0.013276	0	0
UCG002	0.00329059	0	0	0	0	0	0
Unclassified	0	0	0	0	0.001702	0	0
Veillonella	0	0	0.0424373	0.0004539	0.001475	0	0.006014
Vibrionimonas	0.00317713	0.000680812	0.0005673	0.0039714	0.003404	0	0.014978
Xanthobacterace	0.00011347	0	0.0003404	0.0004539	0.000454	0	0.000113
Clostridiuminnoc	0	0	0.0021559	0	0.000454	0	0
Eubacteriumcop	0.00578691	0.000453875	0.0087371	0	0	0	0
Eubacteriumhalli	0.01940315	0.100306366	0	0	0.000227	0.00034	0
Eubacteriumvent	0.00011347	0.006354249	0	0	0	0	0
Ruminococcusgr	0.00045388	0.040394871	0.0723931	0.0052196	0.051061	0	0
RuA143A180min	0.00794281	0.024849654	0	0	0	0	0

OTU ID	FC	JJY	WYX	YYB	QBL	YCS	YT
Acidaminococca	0	0	0.0487916	0	0	0	0
Alicyclobacillales	0	0	0	0	0	0.008851	0
Bacillales	0.01225462	0.001361625	0.021786	0.0011347	0.002269	0.206059	0.010099
Bacteroidales	0.00453875	0.020651311	0.2427096	0.0163395	0.088619	0.000681	0.816635
Bifidobacteriales	0.0493589	0.088959492	0	0.1662317	0.198457	0.001816	0
Burkholderiales	0.02042437	0.001588562	0.059798	0.0029502	0.005106	0.387155	0.014524
Caulobacterales	0.00102122	0	0.0049926	0.0001135	0.00034	0.062975	0
Chitinophagales	0.00442528	0.000680812	0.001702	0.0061273	0.003971	0	0.019063
Chloroplast	0	0	0.003631	0	0	0.019744	0
ClostridiaUCG01	0	0	0	0	0.000227	0	0
Clostridiales	0	0	0	0.0182685	0	0	0
Coriobacteriales	0.014524	0.00090775	0.0154317	0	0.001589	0	0
Corynebacteriale	0.00011347	0	0	0.0003404	0.000454	0	0.001475
Enterobacterales	0.00283672	0.025757404	0.1241348	0.0004539	0.000227	0	0.009531
Erysipelotrichale	0	0.001928969	0.0130489	0.0014751	0.001021	0	0
Eubacteriales	0	0	0	0	0.025417	0	0
Lachnospirales	0.22705095	0.781232271	0.2210371	0.0180415	0.167593	0.001362	0
Lactobacillales	0.01429706	0.002382844	0.0502667	0.7306252	0.324294	0.000454	0.086123
Micrococcales	0.00022694	0.000680812	0.0040849	0.005333	0.000908	0.026438	0.000681
Monoglobales	0.00295019	0	0	0	0	0	0
Oscillospirales	0.57244979	0.058095995	0.0408487	0	0.162714	0.003631	0
Peptostreptococ	0.02121865	0.010666062	0.0169068	0.0052196	0.000113	0	0.018722
Propionibacteria	0.00022694	0	0.0006808	0	0.000227	0.002042	0
Pseudomonadal	0.00068081	0.000113469	0	0.0007943	0	0	0
Reyranellales	0	0	0	0	0.000454	0	0
Rhizobiales	0.00397141	0.003290593	0.0078293	0.0170203	0.004198	0.004766	0.015205
Rubrobacterales	0.00011347	0	0.0002269	0	0	0.003291	0.000113
Sphingomonada	0.00714853	0.000794281	0.0369908	0.0007943	0.001475	0.270056	0.001362

Staphylococcale	0	0	0	0	0.000227	0.000681	0
Unclassified	0	0	0	0	0.001702	0	0
VeillonellalesSele	0.01747419	0	0.054465	0.0006808	0.001475	0	0.006014
Verrucomicrobia	0.00851016	0	0	0	0	0	0
Xanthomonadale	0.01418359	0.00090775	0.0306366	0.0080563	0.006922	0	0.000454

OTU ID	fangchen	jujingyi	wuyexing	yangyanbo	qianbinlin	youchang	yutao
Aeriscardovia_Ur	0	0	0	0	0.178713	0	0
Agathobacter_Ur	0.00204244	0.013162374	0	0	0	0	0
Akkermansia_mu	0.00851016	0	0	0	0	0	0
Anaerostipes_ca	0	0	0.008964	0	0	0	0
Anaerostipes_ha	0.00102122	0.154317486	0	0	0	0.000454	0
Anaerostipes_un	0	0.000340406	0	0	0	0	0
Asinibacterium_u	0.00045388	0	0.0002269	0.0001135	0	0	0.000113
Bacillus_uncultur	0.00011347	0	0	0	0.000454	0.000681	0.000227
Bacteroidesdorei	0	0.000453875	0	0	0.005106	0	0
Bacteroidesdorei	0	0	0	0	0.000567	0	0
Bacteroidesfragil	0	0.001361625	0.0024963	0.0115738	0.001929	0	0.026665
Bacteroidesfragil	0	0.015658686	0.165097	0.0047657	0.011233	0	0.789856
Bacteroidesovatu	0.00022694	0.000340406	0	0	0.001362	0	0
Bacteroidessalye	0	0	0	0	0.001589	0	0
Bacteroidestheta	0	0	0.0004539	0	0.002837	0	0
Bacteroidestheta	0	0.000113469	0.0436855	0	0.003177	0	0
Bacteroidesunifc	0.00022694	0.001248156	0.0308635	0	0.017474	0.00034	0.000113
Bacteroidesvulga	0.00147509	0.000567344	0	0	0.012028	0	0
Bacteroidesvulga	0.00249631	0.000453875	0	0	0.019744	0.00034	0
Bacteroides_Unc	0	0.000226937	0	0	0.000681	0	0
Bacteroides_unc	0.00011347	0.000226937	0	0	0.003971	0	0
Bacteroides_unc	0	0	0.0001135	0	0.000681	0	0
Bifidobacteriace	0	0	0	0	0.000227	0	0
Bifidobacterium	0.04379893	0	0	0	0	0	0
Bifidobacterium	0	0	0	0.0001135	0.009077	0	0
Bifidobacterium	0	0	0	0.0097583	0	0	0
Bifidobacterium	0	0	0	0.0121412	0	0	0
Bifidobacterium	0.00555997	0	0	0.0538977	0	0	0
Bifidobacterium	0	0.088959492	0	0.0903211	0	0.001816	0
Bifidobacterium	0	0	0	0	0.001702	0	0
Bifidobacterium_	0	0	0	0	0.008737	0	0
BlautiahanseniiD	0	0	0	0	0.000227	0	0
Blautia_Unclassif	0.0018155	0.000567344	0	0	0.052876	0	0
Blautia_uncultur	0.05934415	0.111085896	0.1057529	0	0.002723	0	0
Blautia_uncultur	0.00590037	0.008396687	0.0013616	0	0	0	0
Bradyrhizobium_	0.00056734	0.000340406	0.0021559	0.0022694	0.001135	0.004539	0.004993
Butyricicoccus_U	0	0	0	0	0.002269	0	0
Caulobactersp	0.00090775	0	0.0038579	0.0001135	0.000113	0.026438	0
Caulobactervibri	0	0	0.0011347	0	0.000227	0.005673	0
Caulobacteracea	0.00011347	0	0	0	0	0.030863	0
Chitinophaga_Ur	0.00079428	0	0.0009078	0.0020424	0.000567	0	0.003971
Citrussinensissw	0	0	0.003631	0	0	0.019744	0
Clostridiumsensu	0	0	0	0.0182685	0	0	0
Collinsellaaerofa	0.014524	0	0	0	0	0	0
Comamonasaqu	0.00011347	0	0	0	0	0	0
Cutibacteriumac	0.00022694	0	0.0006808	0	0.000227	0.002042	0
Delftiaacidovora	0	0	0	0	0	0.004766	0
DialisterinvisusD	0	0	0.0062408	0	0	0	0
Dialisterpneumo	0	0	0.0057869	0.0002269	0	0	0
DialisterspMarse	0.01747419	0	0	0	0	0	0
Doreaformiciger	0.00397141	0.017587655	0	0	0	0	0

Dorea_unculture	0.00816975	0.039487121	0	0	0	0	0
Eggerthella_Uncl	0	0.00090775	0.0154317	0	0.001362	0	0
Enterococcusaviu	0	0	0.0031771	0.0095314	0.003291	0	0
Enterococcusaviu	0	0	0.0006808	0.0032906	0.002496	0	0
Enterococcusfae	0	0	0	0.0009078	0.000454	0	0
Enterococcusraff	0	0	0.0005673	0.0006808	0.000681	0	0
Erysipelatoclostri	0	0	0	0	0.000227	0	0
Erysipelatoclostri	0	0.001928969	0.010893	0.0014751	0.00034	0	0
Erysipelotrichace	0	0	0.0021559	0	0.000454	0	0
Escherichiacoli	0.00011347	0.001248156	0.0002269	0	0.000227	0	0.000113
Escherichiamarm	0.00272325	0.02428231	0.1232271	0.0004539	0	0	0.006241
EscherichiaShige	0	0.000226937	0	0	0	0	0
Eubacteriumcalla	0	0	0	0	0.02519	0	0
Eubacterium_Un	0	0	0	0	0.000227	0	0
Faecalibacterium	0.10734143	0.006467718	0.008964	0	0	0	0
Faecalibacterium	0.08033587	0.020083967	0.0204244	0	0	0.002156	0
Faecalibacterium	0.02643822	0.000113469	0	0	0	0.000681	0
Flavonifractorpla	0	0.000453875	0.0009078	0	0.148417	0	0
Flavonifractor_Ur	0	0.000113469	0	0	0.005333	0	0
Flavonifractor_ur	0	0	0	0	0.001929	0	0
Fusicatenibacter_	0.06286168	0.174741859	0	0	0	0	0
Fusicatenibacter_	0.00680812	0.013162374	0	0	0	0	0
Granulicatella_ur	0.00022694	0.000226937	0	0.0037445	0.000227	0	0.003177
Howardella_Uncl	0	0	0	0.0080563	0	0	0
Hungatella_Uncl	0	0.001248156	0	0	0.003291	0.00034	0
Hydrogenophilus	0	0	0	0	0	0.00034	0.002269
IncertaeSedis_un	0	0	0	0	0.001021	0	0
IncertaeSedis_un	0	0.00090775	0.0009078	0	0.003744	0	0
Intestinibacterba	0.00317713	0.006127312	0.0007943	0.0032906	0	0	0
Klebsiellaaeroge	0	0	0.0006808	0	0	0	0.002042
Klebsiella_Unclas	0	0	0	0	0	0	0
Labrys_Unclassifi	0	0	0	0.0002269	0.000227	0	0.000227
Lachnoclostridiu	0	0	0	0	0.027346	0	0
Lachnoclostridiu	0.0018155	0	0	0	0	0	0
Lachnoclostridiu	0	0	0	0	0.000227	0	0
Lachnospira_Unc	0.00022694	0	0	0	0.000227	0	0
Lachnospira_unc	0	0	0	0	0.000227	0	0
Lachnospiraceae	0	0	0	0	0	0	0
Lachnospiraceae	0.00011347	0.000226937	0	0	0	0	0
Lachnospiraceae	0	0.005446499	0.0263247	0	0	0	0
Lachnospiraceae	0	0	0	0	0.011687	0	0
Lactobacilluscase	0	0	0	0.0012482	0	0	0
Lactobacilluscris	0	0	0	0	0.006354	0	0
Lactobacilluscris	0	0	0	0	0.047203	0	0.000227
Lactobacillusfern	0	0	0.0001135	0.0157722	0	0	0
Lactobacillusgall	0	0	0	0	0.033473	0	0
Lactobacillushelv	0	0	0	0	0.002042	0	0
Lactobacillusjohr	0	0	0	0	0.007489	0	0.000227
Lactobacillusjohr	0	0	0	0	0.001475	0	0
Lactobacillusjohr	0	0	0	0	0.000227	0	0
Lactobacilluspro	0	0	0.0001135	0	0.035629	0	0.000681
Lactobacillusreut	0	0	0	0	0.002042	0	0
Lactobacillussaliv	0	0	0.0054465	0.4870078	0.110745	0	0.00034
Lactobacillussaliv	0	0	0	0	0.000227	0	0
Lactobacillus_Un	0	0	0.0001135	0.0002269	0.043005	0	0.000681
Lactobacillus_un	0	0	0	0	0.000794	0	0
Lactobacillus_un	0	0	0	0	0.005333	0	0
Lawsonellaclevel	0	0	0	0	0.000113	0	0

Ligilactobacillus_	0	0	0	0.00556	0.006241	0	0
Limosilactobacill	0	0	0	0	0.000908	0	0
Mesorhizobium_	0.00079428	0.000567344	0.0023828	0.0041983	0.001021	0.000113	0.003291
Methylovirgula_U	0.00022694	0.000226937	0	0.0026098	0.000681	0	0.001929
Microbacteriuml	0	0	0	0.0001135	0.000454	0	0.000113
Microbacterium_	0.00011347	0.000680812	0.0040849	0.0052196	0.000454	0	0.000567
Monoglobuspec	0.00295019	0	0	0	0	0	0
Mycobacteriumr	0.00011347	0	0	0.0003404	0.00034	0	0.001475
Olsenellasporalta	0	0	0	0	0.000227	0	0
Parabacteroidesc	0	0	0	0	0.004312	0	0
Parabacteroidess	0	0	0	0	0.001929	0	0
Paraburkholderia	0.00034041	0	0	0.0006808	0.000794	0	0
Pediococcusacid	0	0	0	0	0.009304	0	0
Peptostreptococ	0	0.000113469	0.0040849	0.0001135	0.000113	0	0.018722
Phascolarctobac	0	0	0.0487916	0	0	0	0
Phyllobacteriumi	0.00170203	0.002155906	0.0029502	0.007262	0.000227	0	0.004425
Pseudarthrobact	0	0	0	0	0	0.014637	0
Pseudolabrys_Ur	0	0	0	0	0.000227	0	0
Pseudomonaspu	0.00068081	0.000113469	0	0.0007943	0	0	0
Ralstoniainsidios	0	0.000113469	0.0003404	0.0002269	0	0.008397	0.000567
Ralstoniapicketti	0.01985703	0.001475094	0.0587768	0.0020424	0.003858	0.366844	0.011233
Ralstoniapicketti	0.00011347	0	0.0006808	0	0.000113	0.005673	0.00034
Ralstonia_uncultu	0	0	0	0	0	0.001135	0.000113
Renibacteriumsa	0.00011347	0	0	0	0	0.011801	0
Reyranella_Uncla	0	0	0	0	0.000454	0	0
Rhodanobacter_	0.00703506	0.000113469	0.0031771	0.0066947	0.003404	0	0.000454
Rhodopseudomo	0.00056734	0	0	0	0.000227	0.000113	0.000227
Romboutsia_unc	0.01804153	0.004425281	0.0120277	0.0018155	0	0	0
Roseburia_uncul	0.0417565	0	0	0	0	0.000227	0
Rubrobacter_Un	0.00011347	0	0.0002269	0	0	0.003291	0.000113
Ruminococcusfa	0.00794281	0	0	0	0	0	0
Ruminococcus_U	0.00692159	0	0.0002269	0	0	0.000113	0
Ruminococcus_u	0.0348349	0	0.0006808	0	0	0.000113	0
Salmonellaenteri	0	0	0	0	0	0	0.001135
Sphingomonasle	0.00714853	0.000794281	0.0366504	0.0007943	0.001248	0.24827	0
Sphingomonassp	0	0	0.0003404	0	0	0.021786	0.001362
Sphingomonas_U	0	0	0	0	0.000227	0	0
Sporolactobacilla	0.01214116	0.001361625	0.021786	0.0011347	0.001816	0.205378	0.009872
Staphylococcusc	0	0	0	0	0.000227	0.000681	0
Stenotrophomor	0.00714853	0.000794281	0.0274594	0.0013616	0.003518	0	0
Streptococcusga	0	0	0	0.1700896	0	0	0
Streptococcusgc	0	0	0.0001135	0.0002269	0	0	0.013276
Streptococcusora	0	0	0.0014751	0.0015886	0	0	0.006922
Streptococcuspa	0.00034041	0.000453875	0.0007943	0.0034041	0.000454	0.000113	0.028708
Streptococcussal	0.01304891	0.001475094	0.0344945	0.0056734	0.001589	0.00034	0.005787
Streptococcussp	0	0	0	0.0026098	0	0	0
Streptococcus_U	0.00068081	0.000226937	0.0031771	0.0190627	0.00261	0	0.026098
Subdoligranulum	0.30750028	0.02428231	0	0	0	0.000567	0
Subdoligranulum	0	0.005219562	0	0	0	0	0
Sutterella_uncult	0	0	0	0	0.00034	0	0
Tumebacillus_un	0	0	0	0	0	0.008851	0
Tyzzerella_Uncla	0	0	0	0	0.000681	0	0
Tyzzerella_uncul	0	0	0	0	0.000227	0	0
Tyzzerella_uncul	0	0.000680812	0	0	0.012368	0	0
UCG002_Unclass	0.00329059	0	0	0	0	0	0
Unclassified	0	0	0	0	0.001702	0	0
Veillonellaparvul	0	0	0.0424373	0.0004539	0.001248	0	0.006014
Veillonella_uncul	0	0	0	0	0.000227	0	0

Vibrionimonas_U	0.00317713	0.000680812	0.0005673	0.0039714	0.003404	0	0.014978
Xanthobacterace	0.00011347	0	0.0003404	0.0004539	0.000454	0	0.000113
Clostridiumbolte	0	0.001134687	0.0009078	0.0047657	0.004198	0	0
Clostridiumscind	0	0	0.005333	0	0	0	0
Eubacteriumcop	0	0.000453875	0.0087371	0	0	0	0
Eubacteriumcop	0.00578691	0	0	0	0	0	0
Eubacteriumhalli	0	0.000340406	0	0	0.000227	0	0
Eubacteriumhalli	0.01940315	0.099965959	0	0	0	0.00034	0
Eubacteriumvent	0.00011347	0.006354249	0	0	0	0	0
Ruminococcusgr	0	0.002155906	0.0032906	0.0006808	0.002156	0	0
Ruminococcusgr	0.00034041	0.019403154	0.0487916	0.0027233	0.030296	0	0
Ruminococcusgr	0	0.000453875	0.0196301	0	0.014978	0	0
Ruminococcusgr	0.00011347	0.018381936	0.0006808	0.0018155	0.003631	0	0
Ruminococcusto	0	0.024849654	0	0	0	0	0
humangutmetag	0.00329059	0.067740837	0	0	0	0	0
unidentified	0	0	0	0	0.000227	0	0

Figure3

B

Day	Water+Water				Water+DCA		
0	100	100	100		100	100	100
1	101.6304348	102.8409091	106.547619		107.2626	107.9096	98.31461
2	102.173913	102.8409091	107.142857		108.3799	109.0395	97.75281
3	99.45652174	100.5681818	104.761905		103.9106	105.0847	98.31461
4	101.6304348	102.2727273	103.571429		102.7933	103.3898	99.4382
5	100	100	101.785714		101.676	105.0847	98.31461
6	97.82608696	100.5681818	103.571429		104.4693	106.7797	100.5618
7	98.91304348	101.7045455	109.52381		100.5587	101.1299	95.50562
8	101.0869565	104.5454545	111.309524		101.676	103.9548	97.19101
9	100.5555556	98.8700565	104.597701		97.3262	94.17989	96.64804
10	101.6304348	101.7045455	110.714286		106.1453	105.0847	101.1236
11	105.4347826	107.3863636	117.261905		111.7318	110.7345	105.618

D

Water+Water	Water+DCA	DSS+Water	DSS+DCA
7.9	8.8	6.7	6.3
7.6	7.5	5.8	7
7.8	7.3	7	6.4
	7.7	6.3	5.3
		6.4	6.6
		6.3	
		6.3	

F

Water+Water	Water+DCA	DSS+Water	DSS+DCA
0	0	2	6
0	1	4	5
0		1	6
		3	4

Figure4

	Water+Water	Water+DCA	DSS+Water	DSS+DCA
IL1β	0.987714	0.125381	0.029135	3.182054
	1	0.12084	0.017821	8.29785
	0.143631	0.199808	0.239552	2.121491
		0.044475	0.876812	
IL6	0.03975	2.690189	51.07163	54.90215
	1	5.118585	28.43883	15.7077
	0.305514	0.023373	28.73124	5.897559
		0.259215	34.94834	
			0.618109	

			4.842409	
			4.985135	
IL8	0.639935	1.334741	31.92315	57.4285
	1	0.619074	60.23316	7.537789
	0.019851	0.188087	15.00136	26.12629
		2.37669	*181.6427**	10.34778
			0.277206	0.169583
			0.416618	
			0.129736	
TNF	1.040749	1.371892	19.93777	49.33713
	1	0.927889	30.97444	5.494241
	0.007642	0.665956	13.12375	4.202283
		1.089062	*134.5094**	5.400191
			0.397915	0.046028
			0.059029	
			0.03095	
IL4	1	0.811263	21.30315	22.66882
	1	21.46759	32.50246	23.74419
	1	0.121717	14.58514	36.24243
		1.099767	40.38236	0.820548
			0.056135	15.91162
			43.10091	
			7.932382	
IL10	1	0.92785	20.19917	9.065883
	1	19.76588	*546.6585**	19.60607
	1	0.128278	16.85315	19.90528
		0.822447	11.75159	5.424305
			0.041445	17.50061
			63.11639	
			4.49614	
IL17a	1	0.057996	2.299261	4.5202
	1	1.37375	4.077147	12.20593
	1	0.025811	1.562579	10.69316
		0.085741	22.57373	0.089889
			0.000531	2.194923
			4.167585	
			0.699263	
IFNγ	1	0.01745	1.55112	0.301325
	1	0.00671	0.002967	0.074423
	1	0.011964		

Figure5 A	Water+Water	Water+DCA	DSS+Water	DSS+DCA
CD8	1.37	2.93	2.07	3.99
	2.61	1.88	2.77	2.38
	2.95	1.96	3.5	3.96
				4.88
DC	0.89	1.35	2.26	1.62
	1.09	0	1.66	0.93
	1	0.66	1.29	1.44
				1.4
B220	27.5	44.3	14.3	36.8
	30.4	28.4	28.6	28.8
	49.2	47.5	44.9	39.1
				34.7
NEUTROPHIL	1	0.46	2.84	3.47
	1.46	0.76	1.56	4.77
	0.44		2.01	5.64
				2.11

MONOCYTE	1.31	0.41	3.32	3.59
	0.67	0.65	3.18	4.3
	0.83			3.4
				3.32
MACROPAPHAG	2.71	1.36	6.58	7.46
	2.99	2.12	8.18	7.35
	1.59			8.37
				6.02
CD3	8.11	15.5	11.3	21
	14.2	8.84	10.9	17.1
	13.3	8.86	15.5	19.9
				21.8
CD4	5.75	4.73	5.42	12.62
	8.65	4.6	5.7	9.76
	8.75	5.04	5.69	11.7
				11.18

Figure6
A

OTU ID	DD1	DD2	DD3	DD5	DD6	DW1	DW2
Acidobacteria	0	0.000266205	0	0	0	0	0
Actinobacteria	0.00758685	0.006255823	0.0069213	0.006522	0.007188	0.005191	0.005457
Bacteroidetes	0.08944496	0.112072408	0.0633568	0.0937042	0.136696	0.179822	0.246639
Deferribacteres	0.00039931	0.021030214	0	0.0262212	0.00173	0	0
Deinococcus-Th	0.00066551	0.000133103	0	0.0001331	0.000799	0	0.000133
Firmicutes	0.79102888	0.736323706	0.5996273	0.782111	0.620258	0.761081	0.709703
Proteobacteria	0.10980966	0.121788899	0.3298283	0.0901105	0.232663	0.053507	0.037801
Rhodophyta	0	0	0	0.0001331	0	0	0
Unclassified	0.00106482	0.002129642	0.0002662	0.0010648	0.000666	0.000399	0.000266
Verrucomicrobia	0	0	0	0	0	0	0

D

Tuft cell	Water+Water	Water+DCA	DSS+Water	DSS+DCA
	10.5	5	3.335	3
	5	7.5	2.665	2.335
			11	5.5
			9.5	8
			14.5	
			9	
			13	

Supplementary Figure1

Group	IL-1	IL-6	IL-8	IL-2	IL-4	IL-5	IL-17
A-CD	1.37	177	142	0.05	1.26	0.18	7.28
A-CD	3.46	17.12	77.26	1.52	1.96	1.54	14.41
A-CD	2.39	5.34	225	0.51	1.08	0.72	9.36
A-CD	2.07	21.55	32.48	0.75	1.63	0.71	13.4
A-CD	5.4	5.07	148	2.72	0.53	0.74	4
A-CD	2.44	56.69	90.77	0.92	1.41	0.88	6.37
A-CD	3.24	10.76	230.4	2.2	6.5	1.51	28.57
A-CD	5.38	18.58	86.72	1.75	5.55	1.83	27.99
A-CD	4.44	37.72	155.1	1.77	1.42	1.17	37.46
A-CD	1.77	7.35	20.18	0.54	12.57	0.67	8.78
A-CD	30.14	152.04	806	1.18	0.59	0.98	13.22
A-CD	2.25	8.7	1.14	0.76	0.01	0.89	6.78
A-CD	10.14	460.24	2319.08	1.06	0.45	0.91	3.99
A-CD	4.97	23.62	35.59	3.77	6.65	1.89	22.7
A-CD	5.54	27.44	534	0.98	2.57	0.95	16.9
A-CD	7.02	167.47	635.72	0.68	4.47	1.23	14.08
A-CD	1.38	4.1	16.67	0.92	10.76	1.57	21.78
A-CD	5.82	8.48	513	2.1	5.47	1	10.86

A-CD	1.1	21.85	77.4	0.89	3.02	0.1	11.96
A-CD	2.92	240.3	74.5	1.86	3.34	1.19	48.32
A-CD	30.62	170	667.91	1.32	1.17	0.65	2.56
A-CD	3.88	11.14	33.49	1.19	1.96	1.56	13.75
A-CD	11.14	17.68	167.4	3.8	3.85	1. 01	22.95
A-CD	0.56	6.04	21.95	0.39	0.78	0.01	14.34
A-CD	3.79	10.67	175.18	1.7	1.28	1.47	42.61
A-CD	2.96	20.68	167.2	1.42	2.65	1.47	22.36
A-CD	2.17	1.73	14.35	0.01	0.35	0.01	0.01
A-CD	0.45	4.59	71.4	0.6	0.01	0.01	8.4
A-CD	4.07	283.51	62.72	2.04	5.46	1.15	29.54
A-CD	3.87	5.23	88.79	1.1	2.16	1.47	24.36
A-CD	3.52	22.06	21.39	4.18	5.36	2.06	14.25
A-CD	4.34	82.65	396.37	1.19	1.81	1.04	9.39
A-CD	1.82	9.54	380.8	1.03	0.77	0.93	12.19
A-CD	2.83	13.13	636.33	1.03	0.77	0.93	13.19
A-CD	12.26	18.99	688.88	0.01	0.01	0.01	1.43
A-CD	5.49	25.09	109.5	1.81	4.58	2.09	30.02
A-CD	29.99	71.76	2118	2.17	5.89	0.93	20.48
A-CD	2.41	4.74	88.28	1.89	1.3	0.01	4.88
A-CD	18.49	46.14	1684.13	1.68	0.67	1.78	41.64
A-CD	0.4	23.77	35.37	0.6	1.56	0.01	25.31
A-CD	2.12	8.46	33.65	1.33	3.1	0.19	13.06
A-CD	1.75	55.65	256.32	1.33	0.47	1.43	3.07
A-CD	3.26	14.33	195.68	0.01	0.01	1.69	10.44
A-CD	2.64	13.54	230.67	1.21	0.01	0.01	13.15
A-CD	2.26	24.52	153	0.83	3.57	1.13	28.09
A-CD	3.21	32.65	255.68	0.42	0.02	0.01	6.23
A-CD	8.35	126	652	1.25	2.96	2.46	9.1
A-CD	2.78	2.46	14.69	0.52	4.74	0.91	20.16
A-CD	3.23	11	161	2.58	2.77	1.49	9.77
A-CD	0.78	2.4	9.93	0.73	0.42	0.13	13.15
A-CD	18.7	16.1	54.81	11.96	6.36	2.76	38.18
A-CD	2.44	8.2	34.6	1.2	2.74	1.3	8.01
A-CD	3.51	14.68	26.83	3.52	5.77	2.11	42.43
A-CD	1.04	18.45	13.45	0.82	0.51	1.39	11.5
A-CD	2.85	8.33	234.9	0.07	0.24	0.01	5.35
A-CD	2.85	8.33	234.9	0.07	0.24	0.01	5.35
A-CD	3.71	7.83	93.45	1.57	2.9	1.32	47.59
A-CD	3.16	16.23	10.19	2.19	3.57	1.84	38.72
A-CD	2.08	32.5	101	0.59	2.07	0.16	15.98
A-CD	1.78	89.5	44.33	1.65	1.13	0.01	18.66
Re-CD	1.19	3.11	67.34	1.41	0.18	1.31	8.37
Re-CD	52.61	5.58	129.18	11.84	11.94	0.01	9.98
Re-CD	3.23	12.41	127.63	1.53	0.89	0.22	12.26
Re-CD	0.18	2.89	60.35	0.85	1.98	0.01	14.34
Re-CD	2.44	1.85	29.99	0.91	0.01	0.17	8.37
Re-CD	2.38	2.62	58.9	1.1	0.35	0.17	10.54
Re-CD	1.54	16.06	82.72	0.8	1.69	0.68	0.01
Re-CD	2.31	8.82	22.95	2.91	3.39	1.45	25.16
Re-CD	6.91	13.13	36.01	2.46	2.55	2.52	26.62
Re-CD	3.06	5.52	32.01	2.74	4.44	2.12	26.17
Re-CD	3.08	4.01	169.37	0.59	1.05	0.01	7.39
Re-CD	0.85	1.55	7.69	0.36	0.99	0.27	11.35
Re-CD	10.66	15.02	439.84	3.9	1.12	2.64	19.59
Re-CD	2.41	5.55	251.55	0.64	0.56	0.21	12.9
Re-CD	2.06	6.22	18.3	2.18	3.59	1.79	12.19
Re-CD	20.38	87.42	576.73	1.8	1.3	0.94	27.67

Re-CD	0.01	2.49	1.14	0.36	0.01	0.01	3.45
Re-CD	1.54	5.61	77.78	0.41	0.77	0.64	22.68
Re-CD	3.72	4.26	71.6	1	1.45	0.9	9.44
Re-CD	3.12	9.09	11.62	2.94	3.52	1.65	25.72
Re-CD	5.17	56.17	315	0.76	1.59	1.03	16.52
Re-CD	2.4	6.64	26.84	1.5	6.53	2.15	48
Re-CD	1.42	4.75	54.7	0.39	1.16	0.01	13.13
Re-CD	3.58	4.33	17.33	2.42	6.68	1.63	19.82
Re-CD	7.27	17.14	203.7	2.28	2.5	2.63	18.78
Re-CD	2.13	6.98	117	0.3	0.01	0.59	6.68
Re-CD	6	35.35	464	0.84	0.98	0.96	10
Re-CD	1.87	4.43	80.67	1.13	1.69	0.59	0.01
Re-CD	1.15	4.29	14.79	5.4	6.41	0.01	18.83
Re-CD	1.44	3.99	36.89	0.8	0.01	0.01	25.69
Re-CD	0.88	4.28	30.63	0.47	1.64	0.01	9.59
Re-CD	3.06	10.27	81.16	1.63	5.1	0.97	13.52
Re-CD	6.69	10.67	138	1.25	3.25	0.93	11.4
Re-CD	3	5.05	61.2	1	2.16	0.69	14.7
Re-CD	3.23	12.49	205.98	2.22	1.77	1.23	12.91
Re-CD	3.49	18.27	273.4	0.67	0.52	0.12	4.95
Re-CD	3.49	18.27	273	0.67	0.52	0.15	4.95
Re-CD	1.91	7.22	39.76	0.99	3.59	1.61	19.37
Re-CD	1.39	7.59	32.63	1.3	1.22	0.97	18.83
Re-CD	1.69	2.04	15.35	0.91	0.57	0.01	13.83
Re-CD	0.78	11.49	13.22	0.89	1.04	0.01	11.44
Re-CD	3.23	51.56	252.84	1.17	10.1	1.63	14.66
Re-CD	0.81	0.57	7.28	0.01	0.01	0.01	0.01
Re-CD	1.77	9.12	25.67	0.56	0.75	0.44	20.64
Re-CD	4.00	16.12	16.89	2.63	1.62	1.58	39.51
Re-CD	13.5	11.89	233	5.69	6.16	1.35	23.4
Re-CD	3.65	5.5	67.33	1.91	5.22	1.2	20.83
Re-CD	0.01	1.13	12.16	0.01	0.74	0.45	0.01
Re-CD	2.28	8.54	34.5	2.18	1.77	1.73	22.08
Re-CD	1.49	2.25	146.97	0.99	0.01	1.28	0.01
Re-CD	4.63	8.04	872	1.06	0.61	1.18	19.06
A-UC	4.29	7.56	312	3.15	2.83	1.81	26.62
A-UC	3.57	7.1	68.34	1.33	3.84	2.11	9.39
A-UC	1.78	5.32	40.3	1.4	2.82	2.04	26.16
A-UC	2.09	18.36	67.88	1.39	3.54	2.21	11.77
A-UC	4.23	14.63	332	2.22	3.57	1.96	29.57
A-UC	1.14	6.43	20.8	1.38	2.15	0.79	0.01
A-UC	4.5	18.56	323.69	1.28	2.26	1.81	11.24
A-UC	0.29	2.58	15.51	1.13	1.38	0.01	17.07
A-UC	2.03	20.66	33.83	1.8	5.81	3.9	33.5
A-UC	1.92	6.16	36.66	0.77	2.22	1.44	13.94
A-UC	6.98	63.7	1305.56	2.79	6.94	5.47	18.9
A-UC	3.22	27.77	667	0.63	0.60	0.52	9.09
A-UC	6.74	11.6	54.05	3.62	1.47	1.85	14.71
A-UC	0.02	4.81	22.44	1.09	0.01	0.01	7.61
A-UC	1.67	3.66	12.54	0.72	0.9	1.16	7.24
A-UC	0.01	4.89	46.07	0.52	0.42	0.13	13.55
A-UC	2.18	8.71	89.74	0.56	1.28	0.25	11.87
A-UC	15.89	34.84	506	7.55	0.01	3.4	16.28
A-UC	1.96	4.03	75	2.21	5.84	1.42	17.84
A-UC	1.44	16.17	123.63	0.34	0.01	0.32	8.4
A-UC	3.72	37.1	1077.75	2.29	1.81	1.63	22.98
A-UC	1.06	11.5	120.4	1.59	3.23	1.04	0.01
A-UC	4.93	93.71	241.69	1.5	2.23	0.91	19.43

A-UC	11.35	12.74	763.15	4.9	5.17	2.44	36
A-UC	3.99	14	342	1.94	2.35	2.01	13.97
Re-UC	1.8	4.45	52.08	0.86	1.47	2.16	10.19
Re-UC	3.31	9.01	49.63	1.09	1.19	0.72	16.89
Re-UC	3.52	11.97	31.52	2.3	3.94	1.76	9.98
HC	0.02	3.11	29.02	1.05	2.24	0.23	12.76
HC	1.66	10.17	31.13	1.21	3.02	0.97	16.67
HC	4.44	11.52	56.79	4.84	1.12	2.7	11.83
HC	0.08	1.78	12.93	0.68	2.67	0.01	10.78
HC	6.39	17.45	12.81	4.9	8.08	3.6	41.53
HC	0.01	4.47	5.67	0.73	1.64	0.01	19.84
HC	1.78	5.26	11.84	1.62	4.66	1.46	29.54
HC	0.67	4.28	160.99	0.11	2.45	0.01	9.19
HC	0.57	3.44	8.97	0.45	0.8	0.01	16.2
HC	2.67	5.81	60.4	1.75	1.51	1.16	11.3
HC	1.95	17.11	55.82	0.73	1.38	0.01	12.36
HC	2.18	3.99	197.33	1.41	2.41	0.17	11.57
HC	0.99	4.74	89.53	0.43	5.23	0.01	2.49
HC	1.49	3.94	22.04	1.9	2.89	1.08	22.08
HC	4.58	37.37	1170	0.73	0.33	0.26	9.59
HC	3.07	3.53	83.37	1.48	2.29	0.96	19.2
HC	1.92	3.87	16.2	0.46	0.40	0.69	7.7
HC	14.08	2.6	53.58	13.17	0.01	0.01	8.63

GCDCA	GUDCA	GLCA	TCA	TDCA	TCDCA	TUDCA	TLCA
67.5	2.07	1.24	8.4	0	16.6	0	0.226
478	47.7	40.6	4.3	3.54	12.6	0	0
231	57.2	0.499	41.3	0	165	12.4	0.156
9.89	10.2	12.9	1.16	0	0.78	0.74	0.38
78.2	7.66	0.11	4.19	0	12.4	0	0
255	0	0.457	23.4	0	31.9	0	0.492
68.4	61.3	2.81	0	0	2.97	0	1.48
676	62.9	1.9	32.1	0	184	5.81	0
762	203	0.535	18.3	0	80.1	8.6	0
602	75.3	7.39	36	8.76	46.9	1.48	0.16
108	38.3	3.4	28.7	23.7	58.8	4.08	2.29
968	3.62	0.32	151	0	90.1	0	0
437	0	0.653	9.8	0	64.1	0	0
95.2	136	0.819	4.19	0	25.9	7.09	0.101
598	46.1	0.49	2	0	30.1	1.09	0.31
1910	710	0	29.5	0	268	31.2	0.38
794	144	0	15.4	0	44.1	0	0
1230	151	17.2	16.1	13.6	131	3.85	2.12
592	15.3	0	58.2	0	185	1.87	0
488	89.9	0	0	0	17.4	1.79	0
253	92.5	4.94	2.68	1.68	9.04	0.556	0.59
754	137	1.08	0	15.1	102	1.76	3.86
390	36.9	0	1.12	0	6.98	0.91	0
616	120	0	65.6	0	169	11.9	0
485	186	2.36	12.9	7.49	49.4	6.3	0.127
257	80.8	3.25	14.7	17.9	49.7	4.67	0.648
204	2.88	0	26.4	0	39.1	0.6	0.14
195	0	0	8.34	0	8.13	0	0
403	0	131	15.4	0	68.7	3.43	1.45
229	0	0	27.5	13.5	50.4	0	0
311	29.9	0	37.9	0.246	82.1	3.5	0.12
341	304	1.91	3.09	0.719	24.7	8.41	0.259
253	23	0	13.9	0	39	1.58	0
393	67.8	0.4	4.88	0	21.3	0	0
788	111	2.07	87.4	0	322	9.53	0
285	0	0.108	12.4	0	50.2	0	0.64
530	0	0.817	16.6	0	56	0.402	0.102
758	14.4	0	61	0	231	0	0
0	0	9.29	0	0	1.59	0	0.0352
545	21.1	0	16.4	0	76.2	0	0
287	173	0.595	9.29	0	32.2	5.86	0
97.9	96.7	0.325	1.27	0	4.7	0.989	0.226
103	15.3	1.9	3.84	2.03	13.4	0.242	0.34
1080	22	2.02	8.19	0	54.2	0.09	0
341	140	0	32.7	3.11	44.3	2.64	0
4810	396	0	281	0	446	0	0
1460	184	0.686	93.7	1.76	437	26	0
1050	0	2.45	20.1	0	58.3	0	0
1770	192	0	6.18	0	56.8	1.02	0
293	32	1.15	0.06	0	6.89	0	0
1110	27.2	0	243	0.39	668	7.45	0.39
431	4.99	0	12.2	0	79.6	0	0
1620	0	0.831	29.3	0	75.5	0	0.13
811	54.3	3.48	24.4	22.3	109	2.49	1.73
8140	110	0	163	0	331	1.95	0
901	2.13	0.14	21.6	0	46.1	0	0

155	0	0	9.87	0	15.4	0	0
272	99.5	0.157	6.18	0	13.2	1.36	0
1250	702	0.35	76	0	209	31.6	0
370	0.31	0	#NULL!	0	18.2	0	0.73
1390	72.8	0	12.2	0	44.1	0	0.54
547	25.1	0.995	2	0	9.14	0	0.153
585	37	3.38	13.2	10.2	46.4	0	0
569	9.17	0	14.7	0	49.8	0	0
466	72.3	8.05	8.9	8.93	34.3	1.22	0
612	332	0	0	9.6	38.2	8.91	0
33.9	0	2.29	1.27	0	1.24	0.411	0
1570	0	0	198	0	100	0	0
482	0	0.0847	306	0	53.9	0.907	0.15
511	49	0.24	4.46	0	57.5	0	0.34
888	14.7	0.76	34	0	111	0	0.432
1070	365	1.36	30.6	21.4	109	9.22	0
148	65.9	0	17.8	0	0	1.83	0
72.9	28.8	16.6	0.645	3.73	6.35	0.874	0.688
131	23.8	0	7.76	0	1.91	1.32	0
1070	324	0.482	31.2	0.629	60.8	5.89	0.182
262	240	0	16.3	0	68.7	14.1	0
311	0	0.34	36.5	0	36.3	0	0.153
853	179	0.83	14.5	20.5	118	3.49	1.85
138	84.7	0	2.74	0	8.7	0.86	0
1770	981	36.9	20.5	56	111	18.7	4.33
765	364	99	2.53	21.9	77.5	7.86	4.69
742	274	2.37	9.55	0.829	62.2	8.58	0
214	0	0.535	23	0	27.9	1.03	0.718
215	15.8	1.25	13.9	0	61.8	0.392	0.205
3180	556	0.307	26.9	0	266	23.1	0
691	29.6	0.167	152	3.17	403	8.15	0.0241
531	7.72	0.665	15	0	70.3	1.72	0.0867
916	9.89	0	6.13	0	19.8	1.2	0
447	10.8	0	3.22	0	22.7	0	0
56.3	0	0.591	3.26	0	15	0	0.346
174	8.18	0	4.13	0	27.3	0	0
295	92.1	0.52	26.7	12.6	52	6.05	0.82
688	424	0	25.1	0	103	16.6	0
814	0	7.02	7.13	8.77	37.3	0	0
1590	9.66	2	20.1	0	156	0	0.94
186	60.3	2.03	0	0	11.5	0.2	0
646	341	0	17.1	0	57.2	8.99	0
227	89.2	0	2.35	0	9.74	0.333	0
390	173	0	18.4	8.58	91.2	10.6	0
1370	0	0	39.3	0	128	0	0
1070	923	0.522	10.2	0	73.8	22.8	0.134
1040	130	0	13.5	0	150	7.08	0
488	214	0	17.3	0	46.7	5.71	0
630	422	4	17.2	0	150	37.2	0
285	153	5.34	0	0	0	0.3	0
421	21.7	0	11.3	0	17.5	0	1.38
554	71.4	0	20.3	0	84.1	2.11	0
308	21.5	0	4.85	0	18.3	0	0
202	113	1.06	1.31	0	5.12	0.477	0.399
1150	543	3.12	15	8.21	90	15	0
274	13.9	0	4.21	0	4.86	0.58	0.04
154	123	0	0.85	0	6.67	1.46	0
1950	1190	0	7.02	0	40.4	8.6	0

223	51.2	5.11	2.32	7.23	15.8	1.77	0.995
1470	539	0	13.4	0	62.1	9.27	0
117	13.8	5.11	4.5	4.98	7.47	0	0
6670	628	0.704	27.1	0	255	12	0
4660	177	2.76	140	64.7	732	0	1.52
105	69.8	0	0	0	3.88	0	0.3
89	12.8	0	0	0	1.55	0	0
962	191	0	4.51	0	68	3.87	0
1320	436	0.13	12.6	0	127	17.6	0.64
99920	1710	0	508	0	2360	156	0.15
1210	67.3	3.18	0	0	5.08	0	0
857	445	13.4	19.4	27	14.8	0	0
1640	11.3	0	42.9	0	327	1.22	0
1050	131	0	9.62	0	128	0	0.89
208	57.7	4.58	5.03	9.81	23.9	1.71	0.492
480	26.4	0	5.79	1.26	13.1	0	0.1
495	59.1	3.51	26.6	15.9	123	7.66	1.1
158	79.6	5.18	6.29	9.15	25.2	3.66	0
101	20.1	9.98	6.01	10	10	5	2
1130	74.1	0	54.6	2.84	190	5.35	0.928
3000	72.8	3.08	22.5	0	211	0.51	0
118	49.7	0.675	6.11	4.05	28.9	5.57	0.209
395	120	0.807	12.3	9.01	60.5	5.03	0
791	68.1	5.95	50.6	32.4	145	4.68	1.85
159	3.06	0	16.6	1.06	24.8	0	0.312
469	142	0.718	10.5	2.75	32.2	3.38	0.213
249	52.2	1.38	32.2	6.18	118	9.74	0.3
6470	334	3.19	80	11.1	456	5.66	0
1820	783	0	13.5	0	125	23.7	0
408	62.7	0	4.77	0	57.2	2.28	0
651	21.7	1.51	0	0	65.9	4.61	0
591	19.6	0	20.8	0	53.1	1.05	0
1180	31.6	0	40	0	183	0	0
2850	1410	0.833	26.1	0	305	49.6	0.115
400	184	0	17.3	0	77.1	11.8	0.69
780	16.5	0	33.6	0	139	0	0.18
1190	31.1	0	67.7	0	119	0	0.14
1120	123	0	22.3	0	131	4.51	0
108	16.2	0	2.39	0	15	1.91	0
109	1.32	0	1.95	0	12.2	0	0.31
269	139	3.26	0.97	0	25.5	3.98	0
272	185	1.99	7.61	0.33	24	3.1	0
816	131	0	16.2	0.83	90.7	5.15	0.83
213	13.9	1.34	3.85	1.79	36.6	0	0.257
698	21.8	0.0767	4.8	0	75.7	0.276	0.0498
332	45.9	11.6	31	16.9	46	1.61	1.43
65.4	0	2.05	1.63	5.1	11.5	0	0.157
98.9	37.1	2.9	2.82	1.04	17.1	0.412	0.474
221	66.3	1.39	3.82	3.1	29.3	3.1	0.465
149	22.5	0.199	17.4	0	19.2	2.04	0.647
38.7	0	0.271	3.9	0	18.3	0	0.655
197	132	3.03	4.97	7.44	22.1	4.68	0.834
489	26.7	11.5	9.55	35.4	94.2	1.13	0.77
596	313	0	7.97	0	54.1	8.2	0.178
189	32.1	2.01	10.4	12.3	41.2	0.962	0.191
616	189	3.35	12.6	7.72	87	9.29	0.649
278	27.2	1.25	32.2	47.6	220	7.06	2.92
3130	558	2.59	844	4.13	1700	125	1.17

597	30.8	5.59	19.8	15	69.6	0.593	1.29
359	90	0.136	7.9	1.69	100	9.15	0.542
196	23.2	3.49	1.25	0	11.8	0	0.62
223	81.2	0	6.06	8.37	36.7	3.16	1.36
247	39.6	1.27	59.7	4.7	168	9.17	0.336
938	101	2.96	18.1	0	152	4.25	0.43
1530	463	93.8	52.1	42.9	188	18	3.8
720	19.2	0.767	28.6	3.57	97.2	1.97	0.434
29.8	6.28	4.15	0.65	1.15	10.2	1.38	0.401
167	8.32	4.32	3	6.41	14.6	0	1.22
55.2	7.4	9.34	6.31	7.54	16.6	0.208	0.65
220	94.8	0	8.15	0	58	10.8	0
327	107	7.68	5.56	11.4	60.4	6.88	2.83
473	120	1.81	7.22	0	64.7	2.7	0
328	31.9	0	3.29	0	19.1	0	0
69.4	14.9	13.8	3.36	4.45	11.3	0.346	0.893
1460	266	2.14	20.6	16.5	222	14.7	0.56
822	9.41	0.986	21.8	0	140	0.0333	0.362
1310	396	20.5	10.1	20.9	101	9.71	1.1
3230	228	1.33	115	0	782	22.4	0.928
2830	560	0	186	5.18	570	43.9	0.164
291	59.2	0.535	12.5	13	63.4	2.91	0
753	175	0.916	18.3	0	73.3	4.46	0
312	25.8	4.98	3.48	1.53	15.6	0.206	0.489
464	124	0	14.7	0.491	99.3	8.84	0
1030	418	0	23.6	0	55.9	7.64	0.239
354	98.8	0.648	15.5	0.769	55.2	4.47	0
930	271	16	40.8	46.5	127	13.9	2.19
946	319	0.923	9.57	0	43	5.99	0
117	20.9	21.4	10.2	0	20.9	0	0
743	256	0.737	9.85	0	41.6	4.51	0.0181
829	229	0	14.3	0.57	60.3	6.59	0
495	20.2	0.716	8.48	0	50.9	1.52	0.361
240	112	0.308	7.53	6.92	43.7	6.76	0
1310	15.5	0.246	7.06	4.04	92.4	0.859	0
382	38.3	33.1	7.69	46	91.5	4.23	1.86
117	6.29	0.636	13	0	35.5	0	0.235
579	354	0	7.8	3.75	37.1	6.43	0.33
5210	924	6.94	126	78.8	1160	87.5	2.69
328	90.8	3.96	11.8	6.23	112	5.91	0.803
672	57.9	18.5	11.3	29.7	56.6	0	2.88
1480	425	3.85	7.42	1.42	39.8	3.69	0.159
166	14.5	5.34	8.78	5.1	13.3	0.198	0.364

HC	HC.aver	CD.aver	log2(CD/H	P-value	VIP
115543.7	75475.59	0	-26.16	0.000903	1.1538
183.5525	221.27	132.7	-0.73	0.24735	0.36269
167.122	2491.66	5891.27	1.24	0.98001	0.017944
14338.12	15903.21	27630.58	0.79	0.66758	0.18798
4051.891	8392.5	53614.1	2.67	0.89221	0.11217
13201.94	5422.94	21.63	-7.96	0.000785	1.8058
52627.32	81942.19	77008.3	-0.08	0.32454	0.62271
13371.26	94940.02	89938.66	-0.07	0.60795	0.30431
0	136.92	2360.77	4.1	0.81277	0.16607
7142.325	116258.9	221406.6	0.92	0.89156	0.11226
476077.3	574599.6	9668.15	-5.89	0.00686	2.2941

4820.308	2209.33	19257.85	3.12	0.51506	0.40265
893.08	441.01	14742.81	5.06	0.11676	0.96596
1708.534	637.56	59.99	-3.4	0.10926	0.72308
1108.749	598.9	371.88	-0.68	0.30436	0.40475
55.30652	18.43	0	-14.17	0.21994	0.21174
301.1762	141.67	2623.99	4.21	0.13476	0.83605
25998.5	50794.77	116660.7	1.19	0.64605	0.36245
14202.12	5432.95	11.78	-8.84	0.000752	1.9707
67.5046	30.01	342.19	3.51	0.44912	0.52631
913.2976	366.11	8500.2	4.53	0.42558	0.60416
493.7498	284.59	9870.74	5.11	0.24154	0.86977
2634.299	1006.5	38.13	-4.72	0.048646	1.2618
608.078	202.69	301.64	0.57	0.81069	0.073174
103.7497	64.2	1405.09	4.45	0.22273	0.78602
0	8.55	7.21	-0.24	0.82961	0.057719
69.07215	48.4	1676.84	5.11	0.18255	0.92216
28323.78	51666.18	116340	1.17	0.64684	0.35068
1735222	1278845	2024.8	-9.3	0.00129	2.7264
972.1583	35218.13	1016.05	-5.11	0.23218	0.93793

UC	UC	UC	CD.aver	UC.aver	log2(UC/C	P-value	VIP
20.39068	0	73.98407	2623.99	25.97	-6.65	0.004979	2.6737
0	0	0	1676.84	50.36	-5.05	0.024821	1.5749
663.0265	0	0	77008.3	6893.49	-3.48	0.11569	2.0546
54.51206	22.32199	432.855	1405.09	160.31	-3.13	0.2319	1.2686
313.3093	0	126581.8	116660.7	25588.61	-2.18	0.23593	1.8061
502.3684	0	137963.6	116340	27933.43	-2.05	0.23666	1.7115
0	0	687.464	2024.8	420.81	-2.26	0.25597	0.83111
715.4202	59.0186	3100.375	2360.77	1102.03	-1.09	0.26113	1.2424
360.0177	269.2323	1359.549	19257.85	1025.65	-4.23	0.26783	1.0289
3551.927	0	0	9668.15	710.38	-3.76	0.27522	0.94587
5823.854	332.9525	336.1177	14742.81	1960.1	-2.91	0.2855	0.85678
0	0	16645.18	27630.58	3329.03	-3.05	0.30913	0.58947
0	0	0	342.19	137.27	-1.31	0.34642	0.51498
0	0	0	21.63	0	-14.4	0.34659	0.21689
0	0	110.07	0	22.01	14.42	0.34659	0.21689
0	0	0	11.78	0	-13.52	0.34659	0.21686
0	0	0	7.21	0	-12.81	0.34659	0.2168
0	0	1442.08	0	288.41	18.13	0.34659	0.2169
25.75247	0	197.0389	5891.27	959.09	-2.61	0.38699	1.1587
23.26572	0	75.74914	132.7	84.54	-0.65	0.48195	0.42843
176.8015	0	0	59.99	35.36	-0.76	0.60499	0.22391
65.27054	90.80845	407.2732	8500.2	3122.01	-1.44	0.64278	0.52172
14185.5	0	63328.22	221406.6	86958.78	-1.34	0.66683	0.60049
457.2915	51.66074	2400.899	1016.05	1104.91	0.12	0.67955	0.34316
10714.79	0	69850.78	89938.66	31758.18	-1.5	0.74076	0.28093
491.2494	0	10866.87	53614.1	13541.73	-1.98	0.76989	0.38907
292.1307	0	0	38.13	58.42	0.61	0.87353	0.057482
6303.388	98.36597	726.9554	9870.74	4106.51	-1.26	0.91092	0.11754
0	0	1239.344	371.88	327.02	-0.18	0.91749	0.059251
0	0	991.0929	301.64	349.5	0.21	0.92303	0.045767

UC
0.201634
0.09259
0
0.685351
0.018722

0.001702
0

kind
phylum
phylum
phylum
phylum
phylum
phylum
phylum

kind
class
class
class
class
class
class
class
class
class
class
class
class

kind
domain
domain

kind
family
family
family
family
family
family
family
family

family
family
family
family
family
family
family
family
family
family
family
family
family
family
family
family
family
family
family
family
family
family
family
family
family
family
family
family
family
family
family
family
family
family
family
family
family
family
family
family
family
family
family
family
family

kind
genus
genus
genus
genus
genus
genus
genus
genus
genus
genus
genus

genus
genus
genus
genus
genus
genus
genus
genus
genus
genus
genus
genus
genus
genus
genus
genus
genus
genus
genus
genus
genus
genus
genus
genus
genus
genus
genus
genus
genus
genus
genus
genus
genus
genus
genus
genus
genus
genus
genus
genus
genus
genus
genus
genus
genus
genus
genus
genus
genus
genus
genus
genus
genus
genus
genus
genus
genus
genus

genus
genus
genus
genus
genus
genus
genus
genus
genus
genus
genus
genus
genus
genus
genus
genus
genus
genus
genus
genus
genus
genus
genus
genus
genus
genus
genus
genus

kind
order
order
order
order
order
order
order
order
order
order
order
order
order
order
order
order
order
order
order
order
order
order
order
order
order
order
order
order

order
order
order
order
order

kind
species
species
species
species
species
species
species
species
species
species
species
species
species
species
species
species
species
species
species
species
species
species
species
species
species
species
species
species
species
species
species
species
species
species
species
species
species
species
species
species
species
species
species
species
species
species
species
species
species
species
species

species
species
species
species
species
species
species
species
species
species
species
species
species
species
species
species
species
species
species
species
species
species
species
species
species
species
species
species
species
species
species
species
species
species
species
species
species
species
species
species
species
species
species
species
species
species
species
species
species
species
species
species
species
species
species
species
species
species

species
species
species
species
species
species
species
species
species
species
species
species
species
species
species
species
species
species
species
species
species
species
species
species
species
species
species
species
species
species
species
species
species
species
species
species
species
species
species
species
species
species
species
species
species
species
species
species
species
species
species
species
species
species
species
species
species
species

species
species
species
species
species
species
species
species
species
species
species
species
species
species
species
species

					DSS+Water			
100		100	100	100	100	100	100	100
109.0395		106.7416	107.4866	105.4945	102.6178	104.0936	106.1111	107.5676
109.0395		107.3034	106.9519	105.4945	102.0942	104.6784	106.1111	106.4865
110.1695		102.2472	102.139	104.3956	101.0471	115.7895	102.2222	97.83784
104.5198		102.809	103.2086	101.0989	104.712	107.6023	103.8889	105.4054
106.2147		101.6854	98.39572	103.2967	102.6178	103.5088	103.8889	101.0811
107.3446		100.5618	103.7433	101.6484	98.95288	105.848	103.8889	103.7838
103.9548		93.82022	92.51337	98.35165	96.85864	97.66082	100.5556	97.2973
110.7345		97.75281	97.3262	98.35165	95.81152	98.83041	101.1111	100.5405
102.1053		96.08939	91.23711	91.35135	95.2381	92.26519	97.3262	93.75
111.2994		98.31461	102.6738	95.6044	97.90576	98.83041	104.4444	102.7027
109.6045		102.2472	101.6043	102.7473	105.2356	101.7544	107.2222	108.6486

DW3	DW4	DW5	DW6	DW7	WD1	WD2	WD3	WD4
0	0	0	0	0	0	0	0	0
0.002795	0.00772	0.003993	0.020098	0.007054	0.008519	0.006123	0.018634	0.015706
0.127512	0.225343	0.425263	0.069346	0.060429	0.080926	0.068814	0.045255	0.031678
0	0.022494	0.008918	0	0.000932	0.001597	0.001198	0.001997	0.00173
0	0.000266	0.000532	0	0.000399	0	0.000532	0	0.000532
0.834154	0.63104	0.463596	0.794356	0.860775	0.870491	0.885665	0.894982	0.854519
0.035405	0.112605	0.096899	0.111407	0.06948	0.038067	0.037535	0.038733	0.094902
0	0	0	0	0	0	0	0	0
0.000133	0.000532	0.000799	0.004792	0.000532	0.000266	0.000133	0.000399	0.000932
0	0	0	0	0.000399	0.000133	0	0	0

IL-10	IFNα	TNFα	IFNγ
1.41	0.18	2.74	1.04
3.03	2.77	2.17	5.55
1.78	0.6	2.01	2.38
3.47	2.1	1.84	3.16
3.11	11.96	7.96	1.34
4.22	2.37	2.73	4.57
5.12	3.83	5.37	3.68
6.51	2.21	2.49	6.88
5.47	3.9	4.53	5.18
2.81	2.51	1.35	1.72
3.44	1.42	2.92	2.32
2.01	1.24	1.27	3.27
2.45	5.06	2.31	1.9
4.06	3.83	2.82	7.63
4.14	1.05	2.73	3.01
4.83	2.86	2.03	6.43
3.48	3.48	2.41	3.97
2.52	2.4	2.3	3.42

7.86	1.78	0.79	1.87
3.25	1.27	2.28	3.84
2.08	1.61	1.24	1.96
3.69	2	1.65	2.74
2.6	7.65	7.44	5.65
3.05	0.01	1.51	3.63
3.755	0.84	2.01	2.84
3.27	2.3	2.38	3.54
3.52	0.01	1.13	1.02
1.93	2.43	2.98	0.8
21.42	1.99	2.04	3.53
3.91	2.71	3.51	4.39
5.11	3.78	5.3	5.63
3.37	1.65	1.88	4.21
3.44	1.79	4.13	3.51
3.44	1.79	4.13	3.51
1.6	0.01	0.01	1.99
5.56	4.86	3.71	5.23
3.27	3.4	2.05	0.22
3.05	3.77	4.32	2.11
5.69	2.28	3.08	4.15
2.32	1.86	1.27	9.81
3.27	4.03	5.38	3.45
8.49	1.61	2.1	2.72
5.42	1.91	2.69	4.77
2.37	1.36	1.11	1.16
4.37	3.83	4.42	3.9
3.34	1.25	1.55	2.76
4.87	2.77	2.43	4.75
3.39	2.05	2.61	3.74
1.31	2.7	4.49	3.5
3.39	2.74	1.27	5.5
6.6	42.43	31.38	48.16
2.94	2.48	1.97	2.87
8.03	5.33	3.30	10.13
3.39	2.05	1.21	1.37
2.06	0.99	0.33	6.78
2.06	0.99	0.33	6.78
4.76	3.83	7.11	12.54
3.88	2.42	2.67	7.13
4.06	3.49	1.00	3.64
11.62	2.06	2.18	2.98
3.49	2.23	0.98	2.99
1.76	125.8	104.4	3.41
1.47	2.22	1.71	1.7
1.93	1	1.43	3.28
1.03	1.2	2.01	1.2
2.28	1.58	1.49	1.71
2.85	1.36	0.61	1.27
2.94	3.23	2.18	24.02
4.71	9.58	3.64	6.17
4.92	6.08	4.56	7.06
1.91	0.93	0.66	2.44
2.05	1.27	0.43	2.09
7.02	13.1	13.1	5.31
1.88	1.32	2.01	1.32
6.67	60.16	3.16	6.88
4.31	5.84	3.77	4.37

1.87	0.01	0.56	3.3
2.33	0.96	1.21	3.4
2.56	1.41	2.05	1.19
3.44	3.8	3.16	5.63
3.97	2.05	2.67	2.78
5.02	5.7	4.28	3.3
1.56	1.31	0.01	4.21
3.65	3.09	4.84	6.71
3.32	2.68	3.58	3.73
2.61	1.03	10.08	1.67
3.57	1.56	1.88	2.03
2.9	2.55	0.98	1.79
5.18	3.85	4.94	10.94
1.5	1.09	1.67	1.82
2.65	0.01	0.16	0.01
4.07	3.93	3.3	9.22
6.01	2.74	2.87	3.5
2.88	1.96	2.2	2.08
4.12	1.73	1.1	2.9
1.35	1.86	1.58	0.27
1.35	1.86	1.58	0.27
3.69	1.38	2.49	5.01
3.75	2.59	1.19	1.84
5.53	1.47	1.81	1.34
1.99	0.85	1.21	6.04
4.54	3.42	6.12	4.17
01	0.01	0.01	0.01
3.78	1.88	1.24	7.28
5.38	3.73	2.23	5.28
4.51	19.7	17.21	3.77
3.95	3.77	2.45	5.61
1.11	0.01	0.81	0.01
5.3	3.73	3.51	5.01
2.95	1.09	0.98	1.05
3.48	2.1	2.9	3.8
4.5	3.93	5.78	6.17
3.77	2.89	0.99	3.01
4.91	4.28	3.07	4.71
4.04	2.28	3.44	1.95
7.75	3.85	2.06	5.55
3.85	2.06	1.24	2.99
4.14	2.14	2.68	3.4
3.39	2.06	1.69	3.45
107.1	4.28	3.83	4.64
4.42	1.92	2.29	1.5
2.91	5.78	8.2	4.34
2.57	1.56	1.03	1.01
7.86	14.54	9.55	6.46
1.99	2.27	1.35	1.99
4.47	1.75	1.02	2.99
1.93	1.09	2.23	1.16
3.47	1.62	0.91	1.56
5.24	11.43	13.42	4.11
5.29	2.67	3.75	4.81
3.39	1.78	1.03	2.46
25.36	2.35	3.73	9.35
7.56	1.93	2.84	4.59
4.73	1.96	2.21	2.53

10.1	28.9	12.02	6.79
2.47	1.2	5.42	3.42
2.26	1.91	4.11	1.77
1.99	2.97	1.23	4.65
3.18	3.41	3.85	5.72
2.15	1.61	1.51	2.11
3.44	2.29	1.77	2.04
6.06	10.32	7.72	3.59
0.56	0.01	1.19	3.05
6.07	11.79	7.2	6.79
2.54	2.54	2.43	1.8
4.32	2.65	4.42	3.9
1.23	0.5	1.03	2.46
3.46	0.01	1.11	1.88
3.18	1.67	1.22	3.69
6.42	0.01	0.79	2.58
3.39	2.19	2.89	2.81
3.27	0.38	0.95	0.80
3.29	1.44	0.68	4.75
2.76	2.59	2.89	1.76
4.83	2.21	1.71	1.32
2.21	0.9	2.92	2.24
2.95	8.61	7.06	3.62

			DSS+DCA			
100	100	100	100	100	100	100
109.0395	115.8192	106.7039	107.6923	104.3243	106.0109	108.0645
110.1695	116.3842	106.1453	109.3407	104.8649	107.1038	108.6022
105.6497	109.0395	102.7933	103.8462	101.0811	104.3716	104.8387
102.2599	107.3446	104.4693	107.1429	102.7027	106.0109	104.3011
101.6949	105.6497	102.7933	97.8022	95.67568	94.53552	96.77419
102.2599	105.0847	101.676	86.26374	96.75676	92.89617	94.62366
93.22034	97.17514	91.62011	80.21978	92.43243	86.88525	89.78495
94.35028	93.78531	91.06145	76.37363	90.81081	88.52459	87.63441
87.8453	86.55914	82.96703	*0**	96.64804	92.35294	80.68182
89.83051	92.65537	82.68156	*0**	95.13514	89.61749	71.50538
93.78531	97.74011	87.7095	*0**	94.05405	93.98907	*0**

WW1	WW2	WW3
0	0	0
0.020897	0.007321	0.013177
0.074005	0.048582	0.050579
0.000799	0.000932	0
0.000266	0.000133	0
0.850659	0.891255	0.881139
0.052975	0.051644	0.054705
0	0	0
0.000399	0.000133	0.000399
0	0	0
