# Supplementary figures and images for: Deoxycholic acid exacerbates intestinal inflammation by modulating interleukin-1β expression and tuft cell proportion in dextran sulfate sodium-induced murine colitis
Source: PeerJ. 2023 Feb 15;11:e14842. doi: 10.7717/peerj.14842 (PMC9938654; doi:10.7717/peerj.14842)

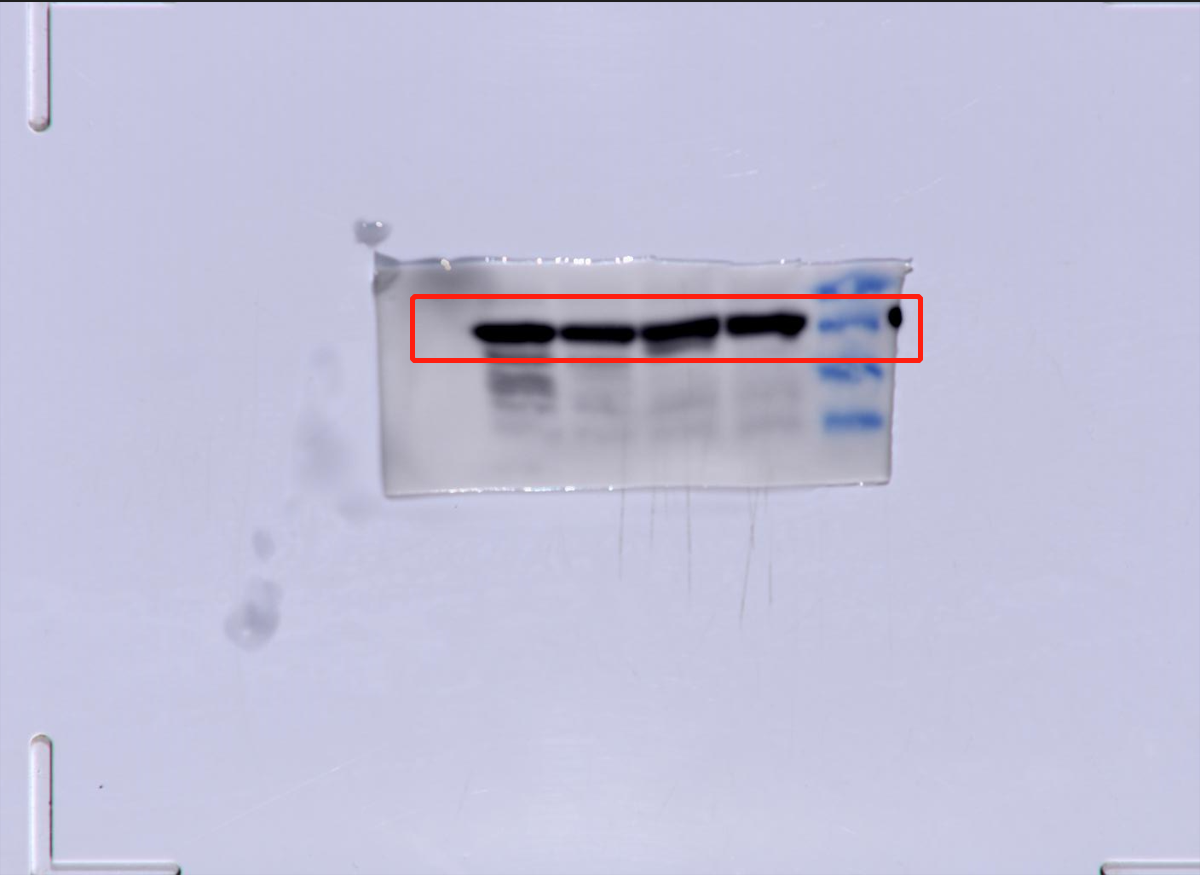

Supplement: Supplemental Information 6 — Beta-actin was the internal control (from right to left: water + water, water + DCA, DSS + water and DSS + DCA). [file peerj-11-14842-s006.png]

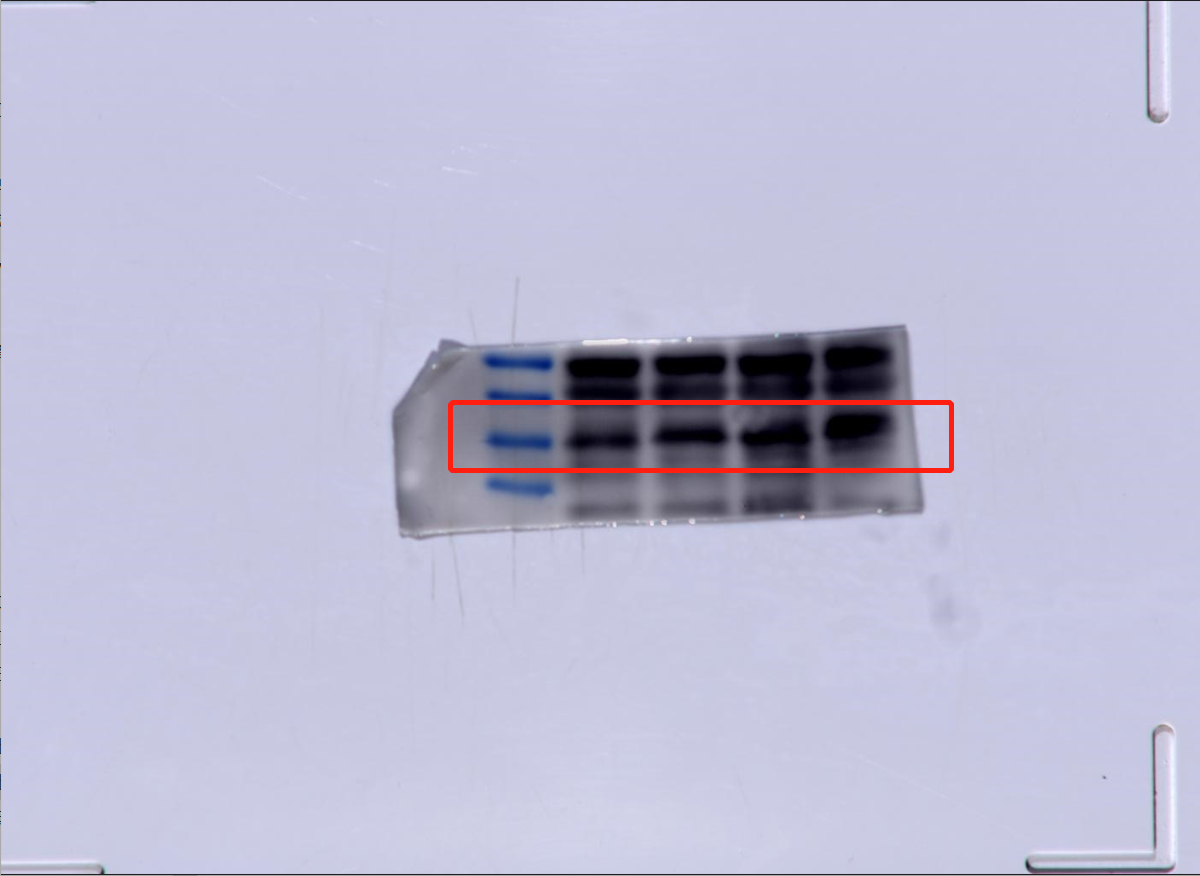

Supplement: Supplemental Information 7 — Total protein was extracted from colon tissues of mice (water + water, water + DCA, DSS + water and DSS + DCA) and IL1beta antibody were used for detection (from left to right: water + water, water + DCA, DSS + water and DSS + DCA). [file peerj-11-14842-s007.png]
